# Trading HIV for sheep: Risky sexual behavior and the response of female sex workers to Tabaski in Senegal

**DOI:** 10.1002/hec.4756

**Published:** 2023-11-02

**Authors:** Henry Cust, Aurélia Lépine, Carole Treibich, Timothy Powell‐Jackson, Rosalba Radice, Cheikh Tidiane Ndour

**Affiliations:** ^1^ Global Health Economics Centre London School of Hygiene and Tropical Medicine London UK; ^2^ University College London Institute for Global Health London UK; ^3^ University Grenoble Alpes CNRS, INRAE Grenoble INP GAEL Grenoble France; ^4^ Bayes Business School, City University of London London UK; ^5^ Division de Lutte contre le Sida et les IST Institut d'hygiène Sociale Dakar Senegal

**Keywords:** condomless sex, economic shocks, female sex workers, HIV, risky sexual behavior, Tabaski

## Abstract

We use a cohort of female sex workers (FSWs) in Senegal to show how large anticipated economic shocks lead to increased risky sexual behavior. Exploiting the exogenous timing of interviews, we study the effect of Tabaski, the most important Islamic festival celebrated in Senegal, in which most households purchase an expensive animal for sacrifice. Condom use, measured robustly via the list experiment, falls by between 27.3 percentage points (pp) (65.5%) and 43.1 pp (22.7%) in the 9 days before Tabaski, or a maximum of 49.5 pp (76%) in the 7 day period preceding Tabaski. The evidence suggests the economic pressures from Tabaski are key to driving the behavior change observed through the price premium for condomless sex. Those most exposed to the economic pressure from Tabaski were unlikely to be using condoms at all in the week before the festival. Our findings show that Tabaski leads to increased risky behaviors for FSWs, a key population at high risk of HIV infection, for at least 1 week every year and has implications for FSWs in all countries celebrating Tabaski or similar festivals. Because of the scale, frequency, and size of the behavioral response to shocks of this type, policy should be carefully designed to protect vulnerable women against anticipated shocks.

## 1 INTRODUCTION

Today female sex workers (FSWs) face 26 times the odds of contracting HIV compared to their female counterparts in sub‐Saharan Africa, up from 13 times in 2018, and are considered a highly vulnerable and key population in the ongoing fight against HIV (UNAIDS, 2018, 2022). In Senegal, HIV prevalence is very low, ≤1%, and credits investments in HIV testing and treatment in keeping prevalence among the general population low (CDC, 2022; World Bank, 2022). However, the prevalence is 19.9% and a concentrated epidemic among FSWs in Senegal (Baral et al., 2012; Kane et al., 2009; Wang et al., 2007). We examine whether Tabaski, an anticipated but largely unavoidable economic shock, leads to risky sexual behaviors in FSWs in Senegal. Eid al‐Adha, or Tabaski in West Africa, is observed as one of the most important annual religious and cultural celebrations in the lunar calendar. The ‘feast of sacrifice’ involves the purchase and ritual sacrifice of a sheep or goat to be cooked and eaten by families and shared with their communities. There is large social and religious pressure to celebrate the quality of their animal as a mark of social status (Aker et al., 2020). In Senegal, around 800,000 animals are sacrificed each year (Sambou & Africanews, 2021), and the most sought‐after animals can fetch as much as €2,200, though typical prices are closer to €200 (Cluzel, 2020). Prices for animals rise dramatically in the days and weeks leading to Tabaski. Animals are not the only costs associated with Tabaski, fabrics, clothing and gifts are additional essential expenses all those celebrating incur.

Unanticipated shocks are typically only unexpected in their timing, but not necessarily in their size and frequency; for example, in the absence of insurance, everyone knows they are likely to become ill during their lifetime with associated health expenses, but predicting when is difficult. Empirically, there is evidence that households in low‐income countries are unable to adequately smooth consumption in the face of large shocks (Gertler & Gruber, 2002). Female sex workers earn a premium for riskier sex acts (Gertler et al., 2005; Rao et al., 2003) and are incentivized to take additional risks during economic hardship. Previous literature finds this premium is relied upon by FSWs and those engaging in transactional sex^1^ to cope with unanticipated economic shocks (Cust et al., 2021). For instance, Burke et al. (2015) find that up to 20% of the cross‐country variation in HIV prevalence is due to droughts in sub‐Saharan Africa, and there are increases in risky behaviors in response to health and civil unrest related economic shocks (Dupas & Robinson, 2012; Robinson & Yeh, 2011). Whilst there is a relatively large literature on the role of shocks on HIV and risky behaviors but no studies of anticipated shocks.

Anticipated shocks, such as Tabaski, differ in one crucial way; there is very little uncertainty. The date is known, and the size is predictable. Given the near‐perfect information, economic theory suggests households would save a portion of income throughout the year to pay for this unavoidable expense, with few real‐life implications^2^ (Deaton, 1991; Friedman, 1957). However, the social and religious pressure to celebrate and the high price of animals means the minimum full participation cost is high, with high social costs associated with partial or no participation. That is, there is a social cost if your family does not celebrate or have an animal to share with the community. Therefore, Tabaski mimics the economic pressure felt from unanticipated shocks. Tabaski also affects entire communities, limiting the utilisation and effectiveness of informal insurance through networks, typically relied upon in lower‐income countries to consumption smooth. There are few policies designed to protect against it and little research on its welfare impacts. This limits the ways in which FSWs can raise money for Tabaski outside of sex work.

This paper studies whether Tabaski elicits similar behavioral responses as unanticipated shocks in the context of commercial sex work. We find inadequate consumption smoothing, with only 10% of FSWs having enough savings to cover the cost of the festival and 62.7% relying on sex as their main method of paying for the costs. Our main finding is that sex acts that took place in the 4–7 days before Tabaski have a condom use prevalence 49.5 percentage points (pp) lower than those that took place more than 23 days before Tabaski, controlling for sex acts in between and recall bias. Those who are yet to buy an animal are unlikely to be using condoms at all, indicating that the economic pressure of Tabaski is likely to be driving risky sexual behaviors in FSWs in Dakar.

Our research design exploits the quasi‐random order of interviews in the third wave of a longitudinal survey of around 600 FSWs to examine the effect of Tabaski on condom use and whether the economic shock or economic pressure^3^ is the mechanism for this result. Sensitivity and social desirability bias mean condom use is over‐reported in our data and is an issue in the risky behaviors literature more widely. We find that when asked directly in our face‐to‐face interviews, FSWs report that 98% of their last sex acts were protected with a condom, and by using these data in our models, the effects of Tabaski were completely hidden. We, therefore, use the list experiment method^4^ to minimize social desirability bias in our outcome and estiamtes.

Our results contribute, first, to the small but growing literature on the effect of religious celebrations as an economic shock and is the first paper to do so in relation to risky sexual behaviors (Aker et al., 2020; Banerjee & Duflo, 2007). Second, our findings contribute to the literature on risky sexual behaviors in response to economic shocks by showing anticipated shocks elicit a similar response as unanticipated shocks (Burke et al., 2015; Gong et al., 2019; Jones & Gong, 2021; Robinson & Yeh, 2011). Third, our study is the first to use the list experiment within observational data seeking to identify a causal relationship in a quasi‐experimental analysis. The significance of this paper is that it sheds light on a novel and important cause of risky sexual behavior in a key population at the focus of the HIV prevention effort. Whilst the prevalence of HIV is lower compared to some countries, Tabaski and other similar religious festivals or holidays occur each year in countries with large FSW populations with higher rates of HIV and therefore, our results are significant for HIV prevention policy around the world.

We start this paper by presenting a conceptual framework to illustrate the decisions and trade‐offs for FSWs in the face of an anticipated economic shock. Second, we describe our data, showing descriptive statistics of the sample and Tabaski. Third, we outline the identification and empirical strategy required for estimating the effect of Tabaski on sexual behaviors. We then present the results of our primary analysis and investigations into the threats and mechanisms, followed by robustness checks. Finally, we discuss the implications and limitations of our results before concluding.

## 2 CONCEPTUAL FRAMEWORK

Our conceptual framework presents the mechanisms through which Tabaski will affect the decision of FSWs and their clients, showing how unprotected sex is used as a means of consumption smoothing by FSWs. We build upon previous frameworks, including Treibich and Lépine (2019) and Gertler et al. (2005).

(1) $$D=f\left(I,U_{\mathrm{client}}\left(R\right)\right)$$

(2) $$S=f\left(P,R^{f},R^{v},A\right)$$

Equation (1) illustrates the demand for sex, *D*, and is made up of clients' disposable income, *I*, and their expected utility, *U*_*client*_, which is a function of the risk, *R*, of the sex act. An important assumption is that men prefer unprotected sex (Randolph et al., 2007). The underlying risk of HIV and other STIs is assumed to be constant, and the impact on preference for condoms is the same for men and women; therefore, cancel out. This leaves the men's preference for protected sex as the key difference driving the positive relationship between price and risk, or the risk premium.

Equation (2) represents the supply for sex where *P* is the price of a sex act, *R* ^*f*^ is the *fixed* risk of working as a sex worker, including the risk of violence and social stigma from each sex act supplied. *R* ^*v*^ is the *variable* health risk for which FSWs have some control. I.e. choice of partners, location of the sex act and condom use negotiation. This risk is positively correlated with price at the sex act level due to the client's willingness to pay to increase with risk and FSWs' disutility of taking health risks. At the time of the study, PrEP was not readily available to FSWs and, therefore, did not enter the conceptual framework and does not pose an issue for our findings. Finally, *A* represents the risk‐coping strategies available to FSWs, in other words, the sources of income or support they can call upon to help smooth their consumption around Tabaski. For example, their savings, additional potential income from a second job, support from clients (outside of sex act related earnings), support from family and friends, or any formal support available from government or NGOs.^5^

(3) $$U_{\mathrm{fsw}}=f\left(H,I\right)$$

(4) $$\frac{\partial I}{\partial R}>0>\frac{\partial H}{\partial R}$$

For FSWs, utility is derived from income, *I*, and health, *H*, Equation (3). Female sex workers maximize utility based on their returns to income and health. Their utility is increasing in income and health. Income is positively associated with risk through the fixed and variable risk, *R* ^*f*^ + *R* ^*v*^, but decreases in health through the risk of HIV, STIs and violence, Equation (4).

(5) $$S=f\left(U_{\mathrm{fsw}}\left(H,I\right),A\right)$$

Finally, since an FSW's income is determined by the price received and their health by the risks taken during sex acts, we can substitute the FSW's utility function into the supply function to give Equation (5). Therefore, the quantity and riskiness of sex supplied by each FSW depend upon their own appetite for risk and the rate at which they can transform health risk into additional income.

Our basic framework allows us to make testable predictions by introducing an economic shock, namely Tabaski. It is a covariate shock affecting the entire community rather than just individuals and has a known impact and date. As the shock nears, the marginal utility of income increases because their discount rate increases. In other words, if we view Tabaski as a challenging savings target, as the time left to reach that target falls, the amount an FSW values income today versus income after Tabaski increases. Female sex workers can raise additional cash from sex work in three ways. First, they can expand supply by increasing the number of hours they work or the number of clients they see. Second, increasing the number of sex acts with each client ‐ leading to an increase in *R* ^*f*^. Third, by negotiating and offering riskier sex, increasing *R* ^*v*^, and charging a relatively higher (or less discounted) price, *P*. Increasing activity on the extensive or intensive margins is difficult when clients will also be considering the upcoming costs of Tabaski. This fall in demand, combined with a potential increase in the supply of sex will lower the average price but have an ambiguous effect on intensity. By offering riskier sex, the price also has upward pressure, again meaning there is an ambiguous impact on price. However, Tabaski's associated demand fall from clients and supply increase from FSWs both work in driving risky behaviors higher in order for FSWs to maintain or increase their income.

A mediating factor is the coping strategies available to FSWs, *A*. Since the shock affects the entire community, including an FSW's network and clients who all celebrate and feel the same economic pressures, it eliminates or reduces the effectiveness of informal coping strategies and resilience from non‐liquid assets (Aker et al., 2020; De Weerdt & Dercon, 2006; Fafchamps, 2010; Townsend, 1995). Debt^6^ and savings are the only effective means of coping with this shock (Deaton, 1991).

## 3 DATA AND DESCRIPTIVE ANALYSIS

### 3.1 Sample

We use the third wave of a longitudinal dataset of FSWs in Dakar, Senegal, that took place from 29th June (32 days before Tabaski) until July 28, 2020 (3 days before Tabaski) with Tabaski on July 31, 2020. The first two waves took place in 2015 and 2017. The third wave, in 2020, was designed specifically to analyze the effects of Tabaski and is the only data used in this paper. The first wave recruited 654 FSWs of 18 old or older living in Dakar using a respondent‐driven methodology, which represented around 15% of the total number of FSWs in Dakar at the time (APAPS & IRESSEF, 2014). Sex work is legal in Senegal if FSWs register with authorities and attend free health check‐ups to confirm they are STI‐free or adhering to antiretroviral drugs. Despite this, around 57% of FSWs in Dakar choose not to register because sex work is morally condemned, and fear of discovery is significantly heightened with registration (APAPS & IRESSEF, 2014; Ito et al., 2018). Registered FSWs were recruited by the midwife in charge of their monthly medical examinations (a registration requirement) from four Dakar sites. Those unregistered, referred to as clandestine FSWs, were recruited from leaders of informal FSW groups, called causerie, that contain both registered and unregistered FSWs. Clandestine FSWs were invited to participate in surveys at the same health centers as the registered. Each participant was given 3000 CFAF (around $5) to cover time and transport costs. In 2020, interviews lasted around 1.5 h and took place at venues near the health centers, taking all measures to minimize COVID transmission.

An objective of the original sample was to analyze registration policy, meaning that around 50% of those recruited were registered, a restriction relaxed in subsequent waves when replacing attrited FSWs (Ito et al., 2018). Replacement in waves two and three were achieved using the same respondent‐driven methodology as wave one, with the proportion of unregistered FSWs now over 53%. Table 1 summarizes the key variables for the sample used in this analysis. The average FSW in the sample is 39 years old and in a household where each adult provides for 1.4 dependents. 37.2% of FSWs earn on average, 51.9% of their income in the last 30 days from their second jobs. This implies the majority of money earned by our sample comes from sex work, with only a few having opportunities to earn significant amounts outside of sex work.

**TABLE 1:** Descriptive sample statistics.

	N	Mean/%	Std. Dev	Min	Max
*Characteristics*					
FSW Age (years)	514	39	9.7	19	63
New respondent to the survey (%)	514	35.8			
Registered (%)	514	46.9			
Gneezy‐Potter risk preference (/2)	514	0.82	0.8	0	2
FSW Interviewed behind schedule (%)	514	9.7			
Time preference (%)	514	80.7			
FSW Household dependency ratio	514	1.4	2.2	0	26
*Economic characteristics*					
Earnings in last 30 days, all sources (CFAF)	512	79,452	99,337	0	1,100,000
Earnings from sex in the last 30 days (CFAF)	512	63,520	86,315	0	900,000
Has savings available tomorrow (%)	514	23.5			
If savings available, quantity (CFAF)	121	208,101	801,941	800	8,000,000
Has a second job (%)	514	37.2			
Non‐sex earnings last 30 days (%)	190	51.9			
Both parents are alive (%)	514	22.4			
Both parents are dead (%)	514	32.1			
Household in debt (%)	509	55.0			
*Highest education level*					
No education(%)	514	51.4			
Koranic education (only) (%)	514	0.8			
Primary education(%)	514	26.1			
Middle school (%)	514	13.0			
Secondary school(%)	514	8.6			
Tertiary education (%)	514	0.2			
*Marital status*					
Never married (%)	514	21.0			
Married (%)	514	0.8			
Divorced or separated(%)	514	70.4			
Widowed (%)	514	7.8			
*Last sex act characteristics*					
Age of last client (years)	511	44	9.6	20	71
Last client was a regular (%)	514	80.7			
Last client has HIV (%)	514	4.1			
Client consumed alcohol (%)	511	10.4			
FSW Consumed alcohol (%)	511	6.5			
Negotiation for price took place (%)	512	46.1			
Last sex in a public place (%)	514	7.6			
Duration of sex‐act (mins)	514	13			
Fellatio took place (%)	513	16.2			
FSW Stayed the night (%)	511	7.6			
Last client was rich (%)	514	5.4			
Self‐reported condom use with last client* (%)	512	97.3			

*Note*: * Condom use prevalence using list randomisation is 65%. Information from Wave 3 only for N = 514, our analytical sample who are active FSWs, we drop those tracked but are no longer active sex workers N = 92. N < 514 due to missing data ‐ refusals and ‘don't know’ responses. Gneezy‐Potter is an investment game to determine the risk aversion of individuals with values of 0 to 2 (Charness and Gneezy, 2010). FSW household dependency ratio is the ratio of children and under 65's to adults in the FSWs household. Time preference is a percentage of those who prefer money today instead of twice as much in one weeks time. Earnings variables are collected by asking FSWs their typical monthly earnings (not reported here but referred to in other sections) and over the last 30 days (reported in the table). Savings variables are defined by asking FSWs if they have savings available to use tomorrow and how much. Non‐sex earnings only for those with second jobs. All last sex act characteristics are as reported by the FSW with the best of their knowledge ‐ ‘Last client has HIV’ equals 1 when the FSW reports a 100% chance the client has HIV.

### 3.2 Tabaski

Of the 514 active sex workers, 83% intended to celebrate Tabaski in 2020.^7^ Overall, FSWs expect Tabaski to cost around 172,000 CFAF (around 311 USD^8^) in 2020. Our sample intended to spend 93,000 CFAF (168 USD) on animals consumed as part of the celebration.^9^ The other festival costs were typically clothes, presents and supplementary food. In the context of our sample, the total personal cost of Tabaski was 121% of a typical month's earnings from sex work, with the personal contribution to the animal costing 67% of a typical month's earnings from sex work.^10^

Purchasing an animal is not straightforward; they are bought alive and must be stored, fed and cared for until the sacrifice. Because many urban households do not have the facilities for storing animals, they must wait until a few days before the feast when large numbers of animals are bought from rural areas to be sold in the city. The prices rise dramatically as a result.^11^

Of active FSWs that celebrate Tabaski, 60% (250 of 418) expect to purchase or contribute to the purchase of an animal, but only 13 of these (5%) had done so at the time of the interview, at a median of 18 days before Tabaski. Of those that have not yet bought an animal, over 90% reported a lack of money being the main reason ‐ not storage problems (5%). Respondents expected to purchase the animal on average 3 days before Tabaski^12^ at a time when prices are likely to be at their peak.

**FIGURE 1:**
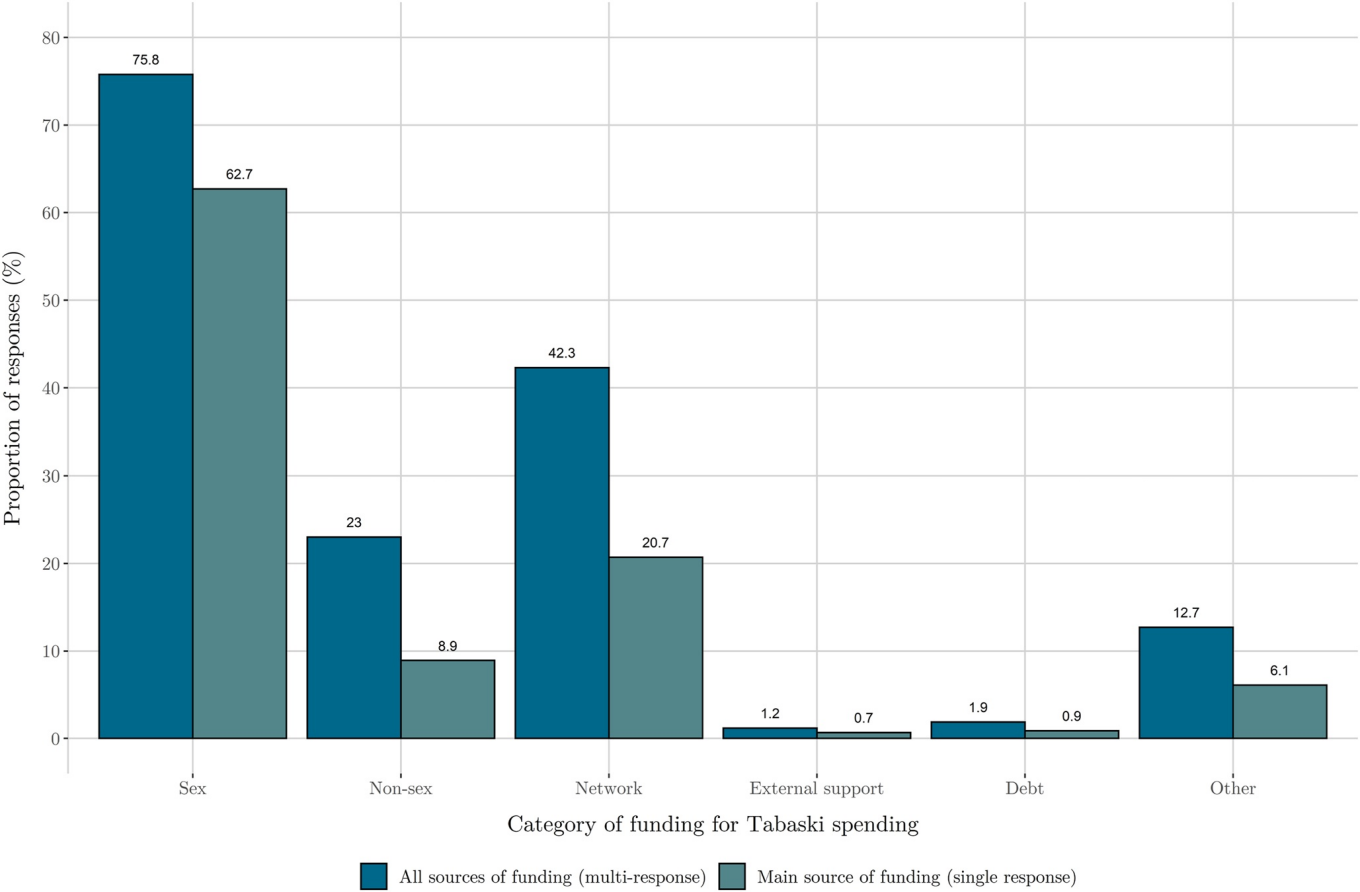
Tabaski funding sources. Bar heights are the percentage of female sex workers (FSWs) that selected each category. ‘All sources of funding’ allowed respondents to tick as many sources as they had contributing to their costs. ‘Main source of funding’ allowed respondents to select only their main source of funding. Only those who are celebrating Tabaski.

Figure 1 shows that the majority of FSWs (62.7%) relied on their sex work to pay for the costs of Tabaski. Only 20.7% of FSWs relied on their networks as the main source of support. The ‘non‐sex’ category contains both savings and income from other sources and was relied upon by fewer than 9% as the main funding source. Less than half of those relying on their network for some of their funding categorized it as their main source of funding, implying resources within networks were scarce. Only 27% have non‐zero savings, with only 10% enough savings to cover their expected cost of the animal. Those with savings do have a high mean level relative to the cost of Tabaski. Data from the second wave in 2017 shows that only 27% had savings available and 11% had enough to cover the average cost of Tabaski,^13^ implying that savings rates are low around this time regardless of COVID.^14^ Low levels of savings are consistent with findings from other low‐income households in Africa (Dupas & Robinson, 2013). If FSWs had enough alternative sources of income and coping depth, that is, sufficient *A* in Equation (2). In that case, our framework predicts the cost of Tabaski would not require taking on additional risks, *R* ^*f*^ and *R* ^*v*^. Table 2 summarizes these data about Tabaski.

**TABLE 2:** Tabaski summary.

	N	Yes	Mean	Proportion
Number celebrating Tabaski in 2020 (n)	514	426		0.83
*Those celebrating Tabaski*				
Intending to purchase an animal* (n)	418	250		0.60
Already bought an animal (n)	250	13		0.05
Total expected cost of Tabaski (CFAF)	426		111,982	
Total expected other costs of Tabaski (CFAF)	426		60,656	
Average number of people celebrating with (n)*	424		9	
*Those celebrating but not purchasing an animal*				
Expected cost when not purchasing an animal (CFAF)	176		37,744	
Earnings in the last 30 days ‐ all source (CFAF)	176		71,316	
Earnings in the last 30 days ‐ sex work only (CFAF)	176		55,833	
*Those intending to but yet to buy an animal*				
How many days before intending to buy animal* (Days)	214		3	
Total expected cost of Tabaski (CFAF)	237		167,110	
Total expected cost of the animal (CFAF)	237		92,468	
Propotion sharing Tabaski costs with at least 1 other (n)	235	108		0.46
Typical earnings over 30 days ‐ all sources (CFAF)	235		169,187	
Typical earnings over 30 days ‐ sex work only (CFAF)	235		139,068	
Earnings in the last 30 days ‐ all source (CFAF)	235		88,536	
Earnings in the last 30 days ‐ sex work only (CFAF)	235		71,921	
Some savings available tomorrow (n)	237	63		0.27
If available, amount of savings available** (CFAF)	63		281,692	
Enough savings to cover expected cost of animal (n)	236	22		0.09

Note: * Missing values due to “don't know” and “more than 1 year in future” being excluded. ** Only those that have savings available.

We asked FSWs about their subjective views on the effect of Tabaski in general on their work. 43% of FSWs who *did not* plan to celebrate Tabaski this year said the number of customers fell, compared to 26% who said they increased. 55% of these same non‐celebrating FSWs said their income dropped, compared to 15% who said it increased, implying that overall, Tabaski depresses the market. Those who *are* planning to celebrate this year were more favorable about Tabaski's effect on the market without tipping the balance overall. However, those celebrating are more likely to be exerting additional effort to earn income, perhaps even displacing the work of those not celebrating. Table 2 shows reduced self‐reported recent earnings compared to typical earnings.^15^

The magnitude of the shock is understated, and it is greater than what is considered a catastrophic health expense, categorized as 40% of monthly expenses after subsistence (Xu et al., 2003). Total Tabaski costs around 138% of total monthly expenditure, with the animal alone costing 75% for Tabaski celebrators. The percentages are 95% and 51%, respectively, accounting for their available savings. For those not purchasing an animal, the costs of Tabaski are still significant, over 50%.

### 3.3 List experiment for condom use

Our primary outcome was condom use during the most recent sex act with a client, measured using the list experiment method. The use of this indirect elicitation method is required given that 98% of FSWs declare to have used a condom during their last sex act when asked directly by an enumerator. This prevalence is 65% when estimated using the list experiment. In the second wave of this survey, direct questioning yielded a prevalence of 97% and 78% via the list experiment (Treibich & Lépine, 2019), implying the use of condoms is a socially desirable behavior and is over‐reported. Previously, the list experiment method has been used for eliciting self‐reported answers for topics including abortion (Bell & Bishai, 2019; Moseson et al., 2021), voting preferences (Gonzalez‐Ocantos et al., 2012; Holbrook & Krosnick, 2010), use of micro‐finance loans (Karlan & Zinman, 2012), opinions on undocumented migrants (McKenzie & Siegel, 2013), gay marriage (Lax et al., 2016) and racism (Krumpal, 2013) and has been shown to be effective to measure condom use (LaBrie & Earleywine, 2000).

The list experiment allows respondents to answer sensitive questions without the fear their answers will be discovered. It minimizes social desirability bias and attenuation bias when estimating marginal effects ‐ a problem in many studies on risky behaviors.^16^ Typically, the list experiment is used to estimate the prevalence of risky behaviors across a sample, but it also allows one to estimate the difference in prevalence between two sub‐groups within a sample. We exploit this to find the difference between FSW's sex acts that are ‘close to’ and ‘far from’ Tabaski as *shocked* and *unshocked* sex acts. We use the validated double list method that improves the efficiency of estimates (Treibich & Lépine, 2019) compared to the single‐sided list experiment detailed in Blair and Imai (2011, 2012).^17^

#### 3.3.1 Implementation

During the survey, when an enumerator reaches the list experiment question, their respondent is randomly allocated to the treatment or control groups for the list experiment by the survey program and asked how many of the following statements the respondent agrees with. It then lists either three non‐sensitive statements for the control group:

• *It is safer to bring a client home than going to the hotel*.
• *I prefer that the client pays me before the sexual intercourse*.
• *Monday is the day I have the greatest number of clients*.

Or for the treatment group, it lists the same three non‐sensitive statements plus a sensitive statement of interest in position 2:

• *It is safer to bring a client home than going to the hotel*.
• *I used a condom during my last intercourse with a client*.
• *I prefer that the client pays me before the sexual intercourse*.
• *Monday is the day I have the greatest number of clients*.

The key assumption is that the average number of non‐sensitive statements agreed with is the same for the treatment and control groups. Therefore the difference in the average number of statements agreed with between each group is the prevalence of condom use at the last sex act.

The double list experiment method simply repeats the list experiment with a new set of non‐sensitive statements and reverses the treatment and control groups allocated in the first experiment. This means over the two experiments; each respondent receives the sensitive statement at least once. The second set of non‐sensitive statements are:

• *The majority of my clients are Senegalese*.
• *I usually spend the whole night with my client*.
• *I usually solicit clients by phone*.

The prevalence can also be estimated using OLS regression analysis. When estimating the prevalence using the double list experiment, each respondent appears in the model as two observations, one when they were in the control group and one in the treatment group of the list experiment. More detail is provided in Section 4.2 of the Empirical Strategy. As you can see, the advantage of this method is that there is no way for the researcher to back out the true answer to the sensitive statement that a respondent has, providing privacy to answer in confidence. This strength is also a drawback since the interpretation of findings can only be made about a group's prevalence and not at the individual level.

#### 3.3.2 Internal validity

The method relies on three key assumptions to be internally valid:

1. Successful randomization of the participants to treatment and control lists.
2. Absence of design effects ‐ the inclusion of the sensitive statement does not change answers to the non‐sensitive statements.
3. Absence or minimization of ceiling and floor effects ‐ the number of respondents who either agree or disagree with all non‐sensitive statements should be minimized to avoid compromising the implicit privacy of the list experiment method.

The validity of this list experiment has been verified in the literature (Lépine et al., 2020; Treibich & Lépine, 2019). In summary, randomization was successful, but there is a chance of a ceiling effect in list B. Since we know the direction of condom use bias is toward under‐reporting, any ceiling effects do not violate privacy and, therefore, do not pose a threat to validity. For completeness, we report the test of the assumptions in Appendix 11.

## 4 EMPIRICAL STRATEGY

### 4.1 Survey design and identification

**FIGURE 2:**
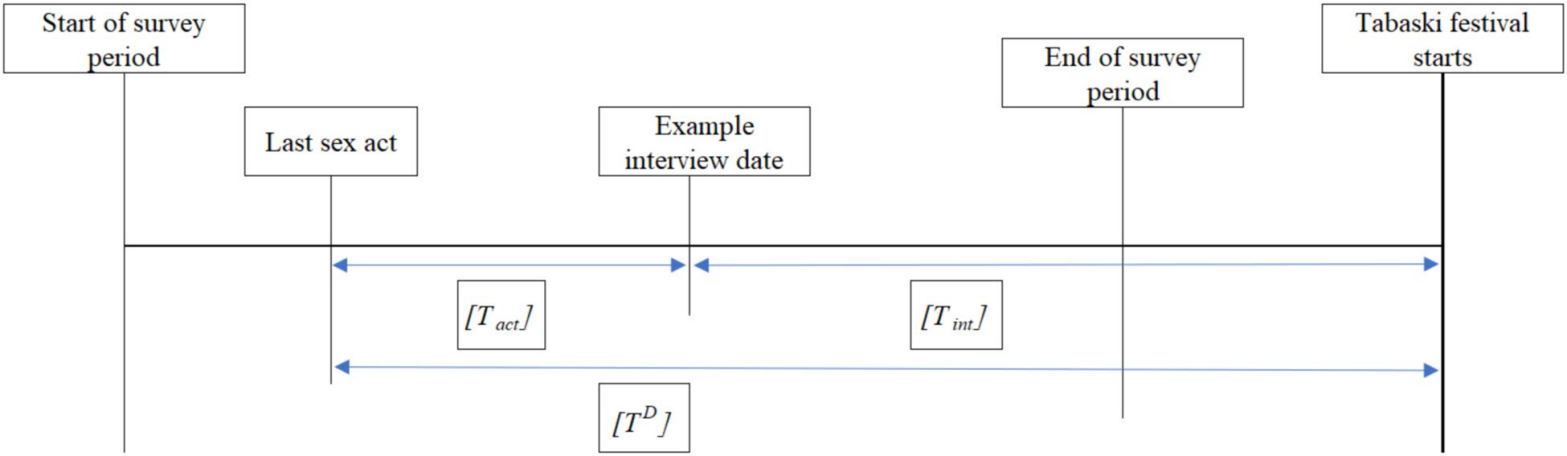
Illustration of key survey dates.

The key variable in our identification of the effect of Tabaski on condomless sex is the number of days between an FSW's last sex act and Tabaski, denoted by *T* ^*D*^. Its determinants are two‐fold, *T* ^int^, the interview date in relation to Tabaski, and, *T* ^*act*^, the time since the last sex act, see Figure 2. Since the analysis is internal, comparing equivalent sub‐samples ’close to’ and ’far from’ Tabaski, we primarily perform an intention‐to‐treat analysis with the whole sample regardless of their personal level of economic pressure felt by Tabaski. We explore differing sub‐samples in Section 5.3.

To ensure the exogeneity of the interview date *T* ^int^, we distributed a list of respondents (those interviewed in wave two) in a randomised order to enumerators. These lists were organized weekly and included spaces for new respondents as replenishment for anticipated attrition of around 30%. Enumerators were instructed to arrange and conduct interviews, moving down the list to arrange interviews at the earliest possible opportunity. Enumerators each received their own list but shared their time to ensure respondents could be interviewed at their earliest preferred opportunity. Should respondents not be interviewed in their allocated week, they were prioritized during the following week's scheduling.

Because the shock is anticipated, and we know that most animal purchases occur in the few days before the sacrifice, we expect the economic pressure to build as this purchase nears, but we do not know if or when behavior change will start to occur. We use *T* ^*D*^ in two ways to determine when an effect occurs and the size of any effect. First, *T* ^*D*^ is used to define a binary variable categorizing sex acts as ‘close to’ or ‘far from’ Tabaski and run individual models for each level of *T* ^*D*^. Second, we use *T* ^*D*^ to define time blocks to be included in the same model.

### 4.2 Estimating equations

**FIGURE A1:**
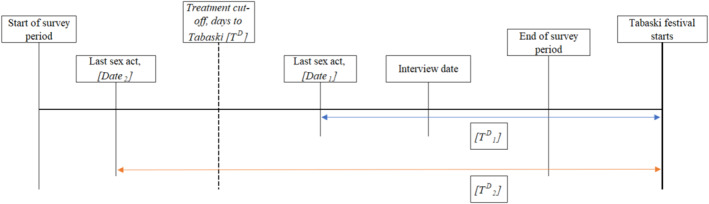
Example of *T* ^*D*^ Categorized to ‘close to’ and ‘far from’ Groups.

We use five equation structures to investigate the impact of Tabaski and its mechanisms. Multivariate analysis of list experiment data is explicated in Imai (2011), Blair and Imai (2012), Moseson et al. (2017) and Lépine et al. (2020). The first specification uses a dummy variable called ‘close to’ Tabaski defined by sex acts within a specific value of *T* ^*D*^. Figure A1 is an example of how a sex act is allocated to a ‘close to’ and ‘far from’ Tabaski. The cut‐off value of *T* ^*D*^ is incrementally changed from four, the lowest value (and closest to Tabaski), to 28 and is estimated by the following equation:

(6) $$LE_{i}=\beta_{0}+\beta_{1}LT_{i}+\beta_{2}T_{i}^{D}+\beta_{3}LT_{i}\times T_{i}^{D}+\beta_{4}List_{Ai}+\beta_{x}^{z}X_{i}^{z}+\beta_{y}^{z}LT_{i}\times X_{i}^{z}+u_{i}$$

Where *LE*_*i*_ is the number of statements that FSW agrees with during the list experiment. *LT*_*i*_ indicates if the FSW was in the list experiment treatment group that included the sensitive condom‐use statement. $T_{i}^{D}$ is a dummy variable that indicates if the last sex act, *i*, was ‘close to’ Tabaski or within *T* ^*D*^, days of Tabaski. $X_{i}^{z}$ is a series of controls variables: ‘being new to the survey’, ‘having a delayed interview’, FSW age and a measure of risk aversion.^18^ *List*_*Ai*_ is a dummy variable indicating if the respondent was a member of the list experiment treatment group for the first or second set of sensitive statements. Because we use the double list experiment, each FSW has two observations, and we estimate robust standard errors clustered at the FSW level to account for this. *u*_*i*_ is the error term across all specifications and is assumed to be independent, with normal distribution, zero mean and constant variance. We vary the value of *T* ^*D*^ from four, its lowest possible value, to 28, estimating a separate regression for each. The coefficient of interest is *β*_3_, which represents the prevalence difference in condom use between those who had their sex act ‘close to’ Tabaski compared to those who had their last sex act ‘far from’, defined by *T* ^*D*^.

Next, we include sub‐groups within this previous specification, Equation (6), to investigate heterogeneous effects and to explore the potential mechanisms:

(7) $$\begin{array}{ll} & \;\;LE_{i}=\beta_{0}+\beta_{1}LT_{i}+\beta_{2}T_{i}^{D}+\beta_{3}G_{i}+\beta_{4}LT_{i}\times T_{i}^{D}+\beta_{5}LT_{i}\times G_{i}+\\ & \beta_{6}T_{i}^{D}\times G_{i}+\beta_{7}LT_{i}\times T_{i}^{D}\times G_{i}+\beta_{8}List_{Ai}+\beta_{x}^{z}X_{i}^{z}+\beta_{y}^{z}LT_{i}\times X_{i}^{z}+u_{i} \end{array}$$

Where *G*_*i*_ indicates a dummy variable equaling one when the FSW is part of the sub‐group in question. *β*_7_ is now our parameter of interest representing the condom prevalence difference between those in sub‐group *G*_*i*_, if their sex act was ‘close to’ Tabaski. Sub‐groups are used to examine differential impacts of time to Tabaski between Tabaski celebrators and non‐celebrations, those that are yet to purchase an animal and those not as well as those in the upper or lower half of the wealth distribution and those with and without available savings.

The advantage of the first specification is that it allows us to pinpoint if there is an effect of Tabaski by varying the definition from four to 28 days. However, this means that as we incrementally change *T* ^*D*^, observations move from the ‘far from’ group to the ‘close to’ Tabaski, meaning we do not have a consistent ‘far from’ comparison group to see how the effect changes over time. The next specification attempts to resolve this by defining a series of time blocks and estimating compared to a fixed reference block in a single regression using the following equation:

(8) $$LE_{i}=\beta_{0}+\beta_{1}LT_{i}+\beta_{2}ListA_{i}+\beta_{c}W_{i}^{b}+\beta_{d}W_{i}^{b}\times LT_{i}+\beta_{x}^{z}X_{i}^{z}+\beta_{y}^{z}LT_{i}\times X_{i}^{z}+u_{i}$$

Where *W* ^*b*^ is a dummy indicating if a sex act occurs in the time block *W* with *b* indicating the block number. *b* = 1 is the block closest to Tabaski, and therefore, the *β*_*d*_ are our parameters of interest, indicating the prevalence difference between these blocks and our reference block. We use two block lengths of four and 7 days, and our reference block is always the block furthest from Tabaski.^19^ The advantage of this specification is that we can see the magnitude of any effect over time with a consistent comparison group.

The third specification gives additional depth and robustness to complement the results from the first two specifications because it dispenses with *T* ^*D*^ as our proxy measure of Tabaski pressure. Instead, we use dummy variables indicating if an FSW is a ‘Tabaski celebrator’ and if they have ‘not yet bought an animal’ as time‐invariant indicators of suffering relatively more economic pressure from Tabaski in the following equation:

(9) $$LE_{i}=\beta_{0}+\beta_{1}LT_{i}+\beta_{2}H_{i}+\beta_{3}LT_{i}\times H_{i}+\beta_{8}List_{Ai}+\beta_{x}^{z}X_{i}^{z}+\beta_{y}^{z}LT_{i}\times X_{i}^{z}+u_{i}$$

Where *H*_*i*_ is a dummy variable of our shock variable of interest. *β*_3_ is our parameter of interest and is interpreted as the condom prevalence difference between those in shock group *H*_*i*_ and those who were not across the whole sample. These shock variables are not exogenous, and results are treated as associative rather than causal.

For outcomes measuring risky sex other than condom use, namely price and client types, which are not subject to the same level of social desirability bias, we can dispense with the list experiment model structure. We also have access to information on both the last and penultimate sex acts for certain outcomes (client type and price of sex act), so we estimate the following models in cross‐sectional (last sex act only) and pooled (both last and penultimate sex acts^20^):

(10) $$Y_{ia}=\beta_{0}+\beta_{1}T_{ia}^{D}+\beta_{2}A_{ia}+\beta_{x}^{z}X_{ia}^{z}+u_{ia}$$

Where *Y*_*ia*_ is the alternative outcome of interest, $T_{ia}^{D}$ is the continuous variable of ‘days between sex act and Tabaski’, and *A*_*ia*_ represents the sex act fixed effect for penultimate sex act *a*. All other assumptions are the same as previous.^21^

### 4.3 Validity

To check the validity of our estimates, we verify that there are no systematic differences in FSWs according to the number of days between the interview and Tabaski since such differences may explain patterns in condom use, see A1.

#### 4.3.1 Date of interview, *T* ^int^

We do however identify two potential threats to the exogeneity of *T* ^int^. First, from ‘new FSWs’ who replace respondents and are answering the survey for the first time in the third wave. The anticipated attrition rate between waves was around 30%, so spaces were left in the lists for new FSWs to be recruited. New respondents were recruited from the network of existing FSWs to maintain the sample of around 600 FSWs. In practice, enumerators did not recruit a uniform number of new FSW respondents across the duration of data collection. Toward the end of data collection, the number of new FSW respondents rises because research teams were prioritizing the continuation of the sample until this point, see Figure A3.

The second threat comes from ‘delayed’ interviews. Not all interviews were conducted when scheduled, with around 10% of respondents interviewed at least 1 week later than their list position. These FSWs were likely to have busy schedules or be less organized, characteristics which could be conceivably linked to their propensity to use condoms and through other unobservables. We include controls for ‘new respondents’ and if the ‘interview was one or more weeks delayed’ to address these potential biases.

Table A1, column 1, in the Appendix, shows the time‐invariant characteristic determinants of ‘date of interview’ relative to Tabaski, *T* ^int^. This confirms ‘new respondents’ and ‘delayed’ interviews are conducted closer to Tabaski but that there are no other significant differences. We explore the potential for bias further in the robustness checks, Section 6, presenting evidence that these variables are unlikely to drive our results as they do not predict condom use when isolated away from Tabaski.

#### 4.3.2 *T* ^*act*^ and recall bias

A third possible source of endogeneity is that the *T*_*act*_ portion of *T* ^*D*^ is not randomly assigned. Ideally, *T*_*act*_ would be sufficiently small in determining *T* ^*D*^ that this imbalance would be trivial. However, the mean *T*_*act*_ is 11.2 days (median 3 days) and mean *T*_int_ is 16.8 days (median 16 days), meaning *T*_*act*_ is skewed away from zero and makes up a large portion of *T* ^*D*^, particularly when *T* ^*D*^ is low. We can view *T* ^*act*^ as a proxy for the frequency of sex acts or the intensity at which an FSW works, which could be related to condom use and bias in our results. *T* ^*act*^ is also likely to be influenced by the proximity of the interview to Tabaski. A complicating factor is recall bias in reporting last sex characteristics. Columns 2 and 3 in Table A1 regress FSW characteristics and last sex characteristics on days since last sex, *T* ^*act*^, showing a relationship with dependency ratio at the 5% significant level and indicators for widows and both FSWs parents being alive at the 10% significance level.

Next, we test for a relationship between *T* ^*act*^, ‘days since last sex’, and condom use to provide evidence of potential unobserved confounding. Since time itself cannot influence the decision to use a condom, any remaining relationship between time and condom use must be via unobservable confounders and recall bias. Table A2 shows no relationship between *T* ^*act*^ and condom use. Since *T* ^*act*^ is a collider, that is, could itself be influenced by Tabaski, we do not include it as a key control variable. We do, however, run versions of our main models, keeping observations where *T* ^*act*^ below a set number. In our robustness checks, we include *T* ^*act*^ and dependency ratio as control variables which our main results are robust to.

#### 4.3.3 Combined

Finally, we combine *T* ^int^ and *T* ^*act*^ to make *T* ^*D*^ to examine its relationship (as a continuous variable) with observables in our data (Table A3). Importantly, combining both does not reveal any new relationships, reassuring that combining *T* ^int^ and *T* ^*act*^ does not introduce new unobserved heterogeneity. Given that some of our estimating equations uses a series of dummy variables to define ‘close to’ Tabaski, see Section 4.2, we also include results from regressions using definitions *T* ^*D*^ ≤ 7, *T* ^*D*^ ≤ 10 and *T* ^*D*^ ≤ 14.

To conclude this section, the evidence we have presented suggests that *T* ^int^ is exogenous conditional on being ‘new to the survey’ or having a ‘delayed’ interview. We include being ‘new to the survey’ as a key control along with having a ‘delayed interview’, ‘FSW age’ as a proxy for experience, and ‘risk aversion’ as it is strongly associated with risky behaviors.

## 5 RESULTS

### 5.1 Main results

**FIGURE 3:**
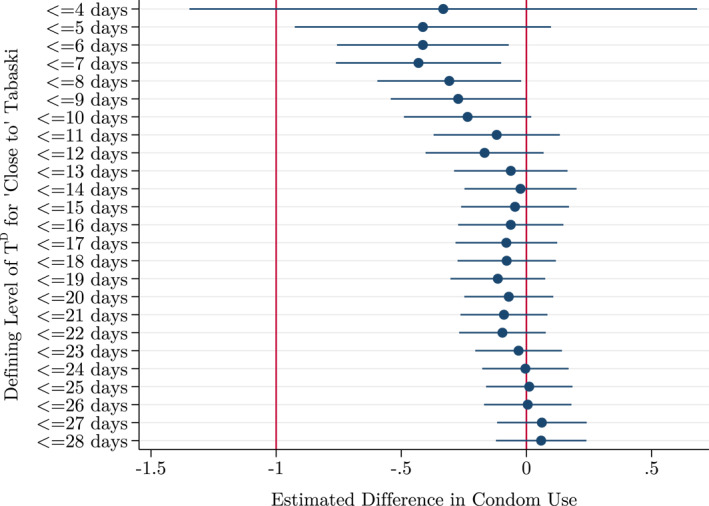
Coefficient Graph of Parameter [*β*_3_] of Models Estimated in Table 3. Includes controls.

**FIGURE 4:**
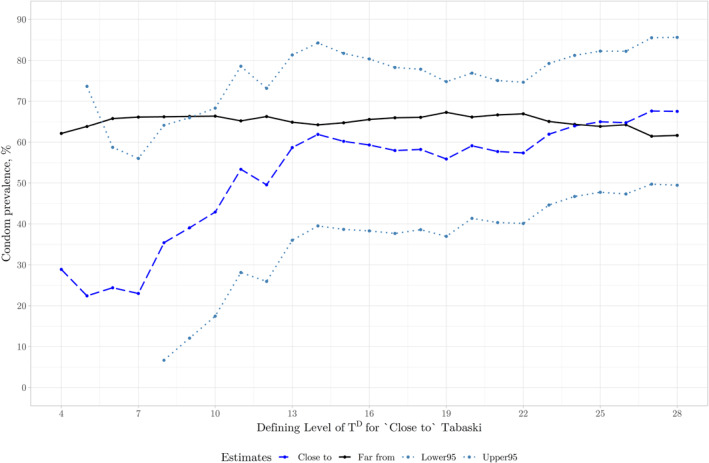
Condom Prevalence with Varying Definitions of ‘close to’ Tabaski. Far from’ Tabaski values estimated as the prevalence of those defined as ‘far from’ Tabaski in specification 1 (Equation (6)) without controls, Table A4. ‘Close to’ Tabaski values estimated using the key parameters, *beta*_3_ in specification 1 (Equation (6)) with controls as well as the 95% confidence intervals.

Our main results are estimated using specification 1 (Equation (6)) with *T* ^*D*^ varying incrementally from four to eleven (Table 3). We find a significant reduction in condom use when we define ‘close to’ as *T* ^*D*^ ≤ 6 to *T* ^*D*^ ≤ 9 inclusive, with a maximum difference in condom use prevalence of 43.1 pp between sex acts within 7 days of Tabaski compared to those 8 days and further from Tabaski.^22^ This implies an approximately 65.5% fall in condom use, to a level of 22.7% for sex acts ‘close to’ Tabaski.^23^ At *T* ^*D*^ = 9, the difference is a 27.3 pp reduction or a 36.6% fall in condom use. The value of *β*_3_ remains negative but non‐significant until *T* ^*D*^ ≤ 24 (see in Figure 3). Figure 4 plots the difference in condom use prevalence across all values of *T* ^*D*^ to 28 calculated using specification 1.

**TABLE 3:** Effect of Last Sex Act being ‘close to’ Tabaski on Condom Use Prevalence.

Variables	(1)	(2)	(3)	(4)	(5)	(6)	(7)	(8)
	*T^D^*	*T* ^*D*^	*T* ^*D*^	*T* ^*D*^	*T* ^*D*^	*T* ^*D*^	*T* ^*D*^	*T* ^*D*^
	< = 4 days	< = 5 days	< = 6 days	< = 7 days	< = 8 days	< = 9 days	< = 10 days	< = 11 days
Close to Tabaski * list	−0.332	−0.414	−0.414**	−0.431**	−0.308**	−0.273**	−0.235*	−0.119
	(0.516)	(0.260)	(0.175)	(0.168)	(0.146)	(0.137)	(0.129)	(0.128)
Close to Tabaski	−0.331	0.086	0.178	0.137	0.160	0.093	0.107	0.018
	(0.248)	(0.171)	(0.121)	(0.119)	(0.114)	(0.107)	(0.102)	(0.100)
Sensitive list	0.337	0.369*	0.349	0.353	0.371*	0.359	0.369*	0.355
	(0.222)	(0.221)	(0.219)	(0.219)	(0.220)	(0.219)	(0.220)	(0.220)
Non‐sensitive list A	−0.349***	−0.349***	−0.343***	−0.342***	−0.341***	−0.341***	−0.341***	−0.342***
	(0.043)	(0.043)	(0.043)	(0.043)	(0.043)	(0.043)	(0.043)	(0.043)
New * list	−0.101	−0.067	−0.026	−0.018	−0.030	−0.032	−0.040	−0.073
	(0.105)	(0.106)	(0.108)	(0.108)	(0.110)	(0.111)	(0.112)	(0.114)
FSW age * list	0.009*	0.008	0.008*	0.008	0.008	0.008	0.008	0.009*
	(0.005)	(0.005)	(0.005)	(0.005)	(0.005)	(0.005)	(0.005)	(0.005)
Risk aversion * list	−0.026	−0.035	−0.014	−0.013	−0.016	−0.019	−0.023	−0.025
	(0.058)	(0.058)	(0.058)	(0.058)	(0.058)	(0.058)	(0.058)	(0.058)
Delayed * list	0.056	0.053	0.066	0.066	0.060	0.066	0.060	0.061
	(0.158)	(0.158)	(0.156)	(0.156)	(0.156)	(0.157)	(0.158)	(0.158)
Constant	2.150***	2.137***	2.135***	2.135***	2.121***	2.132***	2.124***	2.137***
	(0.148)	(0.148)	(0.148)	(0.148)	(0.148)	(0.148)	(0.147)	(0.147)
Observations	824	824	824	824	824	824	824	824
*R* ^2^	0.237	0.236	0.237	0.239	0.235	0.235	0.235	0.233
Double list experiment	Yes	Yes	Yes	Yes	Yes	Yes	Yes	Yes
Key controls	Yes	Yes	Yes	Yes	Yes	Yes	Yes	Yes
*T* ^*act*^ < 90 only	Yes	Yes	Yes	Yes	Yes	Yes	Yes	Yes
Number of FSWs	412	412	412	412	412	412	412	412
FSWs in ’close to’ group	4	17	35	37	48	55	62	68

*Note*: Robust standard errors in parentheses. Specification 1 (Equation (6)) with the last sex act within *T* ^*D*^ days of Tabaski defining ‘close to’ Tabaski. The top row is the parameter of interest, *β*_3_. Each column is a separate regression. Data of double list experiment with FSW level clustered standard errors. The sample is limited to those who have sex acts within the last 90 days, and regressions include the key controls of FSW age, new FSW to the survey, delayed interview and risk aversion. Covariates without list treatment are included but not reported for brevity. There are no sex acts within 3 days of *T* ^*D*^ < = 11 + *days*; the key parameter estimates remain similar and statistically non‐significantly different from zero, see Figure 3.

***p < 0.01, **p < 0.05, *p < 0.1.

**FIGURE A2:**
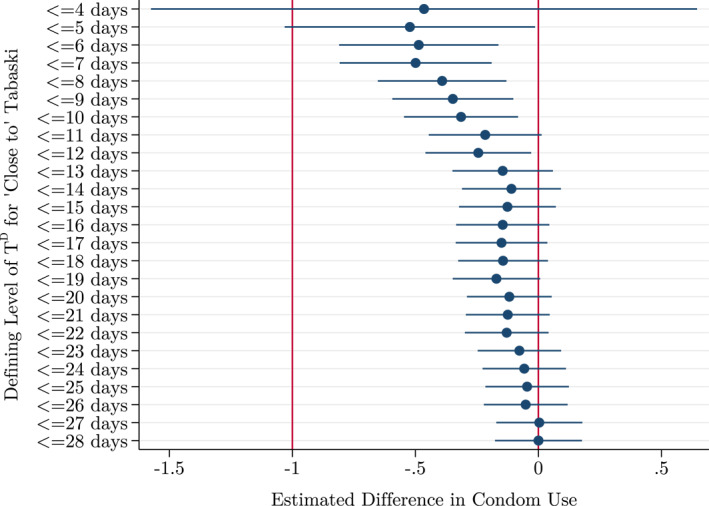
Coefficient Graph of Parameter [*β*_3_] of Models Estimated in Table 3 without controls. Results tables for figures are available on request.

We also present the results from Equation (6) without any controls (Figure A2). Tabaski has a negative effect on condom use, with the absolute magnitude increasing the closer the sex act is to Tabaski. Effect estimates show significant differences between groups when we define ‘close to’ Tabaski as *T* ^*D*^ ≤ 5 to *T* ^*D*^ ≤ 12 inclusive. This implies a maximum difference of 52.2 pp or an 81% drop in condom use when ‘close to’ Tabaski is defined by *T* ^*D*^ ≤ 5. When *T* ^*D*^ ≤ 4, we still find large negative coefficients, but a lack of observations means statistically significant differences are not found at conventional levels. We would expect the effects to persist up to Tabaski or the animal purchase.

The second part of our main results is estimated using specification 2 (Equation (8)) using sex acts grouped into blocks. Table 4 shows a significant drop in condom use for the first block when blocks are sized seven or four. A limitation with models 2 to five is that the range of values *T* ^*act*^ can have in each block varies; therefore, at the cost of observations, we limited the observations in the models by *T* ^*act*^ ≤ *block length*. We find a reduction in condom use prevalence of 47.6 and 49.5 pp for block sizes of seven and four, respectively, for this first block, but no statistically significant effects for any other blocks. In models 3 and 6, this corresponds to a 68% and 76% drop in condom use to a level of 22.6% and 15.4%, respectively, for sex acts within 7 days of Tabaski compared to our comparison block. To test the extent of potential confounding in these models, we perform the robust version of the Hausman test on models 1 and 2, then models 4 and 5, failing to reject the null in both cases, confirming the coefficients of interest are equivalent (Kaiser, 2015; Pei et al., 2019). Finally we include dependency ratio as a key control in place of *delayed interviews* with similar results, available on request.

**TABLE 4:** Effect of Last Sex Act being in Time Blocks on Condom Use Prevalence.

	(1)	(2)	(3)	(4)	(5)	(6)
Variables	Block = 7 days	Block = 7 days	Block = 7 days	Block = 4 days	Block = 4 days	Block = 4 days
Block 1 * list	−0.500***	−0.402**	−0.476**	−0.483***	−0.414**	−0.495**
	(0.162)	(0.175)	(0.197)	(0.163)	(0.179)	(0.212)
Block 2 * list	0.099	0.136	0.068	0.121	0.200	0.213
	(0.116)	(0.124)	(0.149)	(0.140)	(0.151)	(0.193)
Block 3 * list	−0.107	−0.127	−0.174	0.048	0.037	−0.040
	(0.109)	(0.109)	(0.139)	(0.159)	(0.161)	(0.191)
Block 4 * list				−0.201	−0.177	−0.195
				(0.146)	(0.150)	(0.196)
Block 5 * list				0.139	0.101	0.142
				(0.128)	(0.134)	(0.170)
Sensitive list	0.663***	0.335	0.524*	0.646***	0.312	0.215
	(0.058)	(0.220)	(0.271)	(0.060)	(0.223)	(0.286)
Non‐sensitive list A	−0.334***	−0.342***	−0.383***	−0.343***	−0.352***	−0.386***
	(0.043)	(0.043)	(0.055)	(0.044)	(0.044)	(0.058)
Constant	2.058***	2.131***	2.136***	2.076***	2.121***	2.266***
	(0.043)	(0.145)	(0.181)	(0.044)	(0.146)	(0.201)
Observations	826	826	548	826	826	482
*R* ^2^	0.235	0.244	0.248	0.236	0.251	0.249
Prevalence in comparison block	0.663	0.663	0.702	0.649	0.646	0.649
Prevalence in block 1	0.261	0.261	0.226	0.154	0.232	0.154
Double list experiment	Yes	Yes	Yes	Yes	Yes	Yes
Key controls	No	Yes	Yes	No	Yes	Yes
Weekend control	No	No	No	No	Yes	Yes
*T* ^*act*^ < 90 only	Yes	Yes	‐	Yes	Yes	‐
*T* ^*act*^ < = 7 only	No	No	Yes	‐	‐	‐
*T* ^*act*^ < = 4 only	‐	‐	‐	No	No	Yes
Number of FSWs	413	413	274	242	413	242

*Note*: Robust standard errors in parentheses. Specification 2 (Equation (8)) with blocks of seven and 4 days. Block 1 indicates the block closest to Tabaski and all are in reference to the furthest block. Block 1 starts at *T* ^*D*^ = 1. There are no observations between *T* ^*D*^ = 1 and *T* ^*D*^ = 3. ‘Block * list’ are our parameters of interest. Data of double list experiment with FSW level clustered standard errors. Models 1 and 3 are limited to sex acts within 90 days. Models 2 and 4 are limited to sex acts the same as the block length, so reference groups are not over weighted by those with less frequent sex acts. All regressions include the key controls of FSW age, new FSW to the survey, delayed interview and risk aversion. Models 5 and 6 include a control for if the sex act took place at the weekend. Controls and their interacted parameters are not reported for brevity. Prevalence of comparison block is the coefficient on the ”sensitive list” variable in unreported versions of the models that do not include controls. Prevalence in block 1 is the difference between this number and the estimated effect in block 1.

***p < 0.01, **p < 0.05, *p < 0.1.

Both these specifications suggest a strong effect of Tabaski on condomless sex, concentrated in the 7 days before Tabaski with a maximum effect size of between 47.6 and 49.5 pp.

### 5.2 Exploring pathways

#### 5.2.1 Tabaski exposure

We now examine more closely whether the economic pressure from Tabaski is the driving force behind the reduction in condom use using the sub‐group version of specification 1 (Equation (7)). Female sex workers who are financially more exposed to Tabaski, that is, those who have more purchases to make with less support from others and little coping depth, should be more likely to engage in risky behaviors to make up a greater relative amount for Tabaski. First, we construct a dummy variable which takes a value of one if an FSW is celebrating Tabaski and zero if they are not celebrating. As expected, celebrating Tabaski is associated with much lower condom use than non‐celebrators (Table 5). There is a statistically significant difference in condom use prevalence of up to 68.7 pp when ‘close to’ Tabaski is defined as *T* ^*D*^ ≤ 5. The linear combination shows the effect across multiple definitions of ‘close to’ Tabaski, revealing a stronger effect for those celebrating compared to the full sample with a difference of up to 55.3 pp.^24^

**TABLE 5:** Effect of Last Sex Act being ‘close to’ Tabaski differentiated by Tabaski Celebrators on Condom Use.

Variables	(1)	(2)	(3)	(4)	(5)	(6)	(7)
	*T^D^*	*T* ^*D*^	*T* ^*D*^	*T* ^*D*^	*T* ^*D*^	*T* ^*D*^	*T* ^*D*^
	< = 5 days	< = 6 days	< = 7 days	< = 8 days	< = 9 days	< = 10 days	< = 11 days
Celebrate * close to Tabaski * list	−0.687**	−0.513	−0.520	−0.581**	−0.371	−0.319	−0.277
	(0.305)	(0.325)	(0.317)	(0.277)	(0.266)	(0.260)	(0.255)
Close to Tabaski * list	0.187	−0.027	−0.033	0.136	0.012	0.018	0.103
	(0.120)	(0.261)	(0.261)	(0.235)	(0.229)	(0.229)	(0.226)
Celebrate * list	0.051	0.073	0.079	0.109	0.079	0.076	0.073
	(0.114)	(0.120)	(0.120)	(0.123)	(0.126)	(0.127)	(0.128)
Sensitive list	0.323	0.283	0.282	0.273	0.297	0.311	0.304
	(0.247)	(0.245)	(0.245)	(0.247)	(0.246)	(0.247)	(0.247)
Non‐sensitive list A	−0.346***	−0.343***	−0.343***	−0.345***	−0.343***	−0.343***	−0.343***
	(0.043)	(0.043)	(0.043)	(0.043)	(0.043)	(0.043)	(0.043)
FSW age * list	0.008	0.008*	0.008	0.008	0.008	0.008	0.008
	(0.005)	(0.005)	(0.005)	(0.005)	(0.005)	(0.005)	(0.005)
New * list	−0.060	−0.017	−0.009	−0.020	−0.029	−0.039	−0.074
	(0.107)	(0.109)	(0.109)	(0.111)	(0.112)	(0.113)	(0.115)
Delayed * list	0.056	0.073	0.073	0.068	0.072	0.065	0.065
	(0.158)	(0.155)	(0.154)	(0.154)	(0.157)	(0.157)	(0.158)
Risk aversion * list	−0.034	−0.019	−0.017	−0.019	−0.021	−0.026	−0.028
	(0.058)	(0.058)	(0.058)	(0.058)	(0.058)	(0.058)	(0.058)
Constant	2.192***	2.198***	2.194***	2.203***	2.200***	2.194***	2.194***
	(0.169)	(0.170)	(0.170)	(0.171)	(0.171)	(0.170)	(0.170)
Observations	824	824	824	824	824	824	824
*R* ^2^	0.237	0.239	0.241	0.238	0.237	0.236	0.234
Effect of Tabaski on celebrators							
Linear combination	−0.5*	−0.54***	−0.553***	−0.445***	−0.358**	−0.301**	−0.174
*p*‐value	0.084	0.01	0.005	0.008	0.025	0.041	0.233
Double list experiment	Yes	Yes	Yes	Yes	Yes	Yes	Yes
Key controls	Yes	Yes	Yes	Yes	Yes	Yes	Yes
*T* ^*act*^ < 90 only	Yes	Yes	Yes	Yes	Yes	Yes	Yes
Number of FSWs	412	412	412	412	412	412	412
FSWs celebrarting and in ’close to’ group	15	26	28	36	41	48	53

*Note*: Robust standard errors in parentheses. Specification 1 with sub‐groups (Equation (7)) with the last sex act within *T* ^*D*^ days of Tabaski defining ‘close to’ Tabaski interacted with the sub‐group of Tabaski Celebrators. The top row is the parameter of interest, *β*_7_. Linear combination is the effect of being ‘close to’ Tabaski for celebrators. Columns from left to right are separate regressions. Data of double list experiment with FSW level clustered standard errors. The sample is limited to those who have sex acts within the last 90 days, and regressions include the key controls of FSW age, new FSW to the survey, delayed interview and risk aversion. Covariates without list treatment are included but not reported for brevity. There are no sex acts within 4 days for Tabaski for both celebrators and non‐celebrators. Beyond *T* ^*D*^ < = 11 + *days*; the key parameter estimates remain similar and statistically non‐significantly different from zero.

***p < 0.01, **p < 0.05, *p < 0.1.

We next define an alternative sub‐group that is more exposed to Tabaski's economic pressures. We construct a dummy variable that equals one if an FSW had not yet bought an animal but had indicated they intend to. The comparison group includes those who had already bought their animal (N = 13), plus those who had no intention of buying an animal (N = 168), making it a within ‘Tabaski celebrators comparison’. The magnitude of the reduction in condom prevalence between the two groups is large, up to 63.8 pp when we define ‘close to’ as *T* ^*D*^ ≤ 5, but not statistically significant at conventional levels, see Table 6. The linear combinations suggest a stronger and statistically significant effect of being ‘close to’ Tabaski for those who have not yet bought an animal up to 73.8 pp reduction in condom prevalence when the definition is *T* ^*D*^ ≤ 6 and persists to *T* ^*D*^ ≤ 8. Given our comparison group's average prevalence at this definition (Table A4, coefficient on the ‘sensitive list’ variable) is approximately 66%, the effect of having ‘not yet bought an animal’ brings their prevalence to effectively 0%. We find similar findings when we include those not celebrating in the comparison group (*N* = 88), see Table A8.^25^

**TABLE 6:** Effect of Last Sex Act being ‘close to’ Tabaski differentiated by ‘Those still to purchase an animal’ on Condom Use.

Variables	(1)	(2)	(3)	(4)	(5)	(6)	(7)	(8)
	*T^D^*	*T* ^*D*^	*T* ^*D*^	*T* ^*D*^	*T* ^*D*^	*T* ^*D*^	*T* ^*D*^	*T* ^*D*^
	< = 4 days	< = 5 days	< = 6 days	< = 7 days	< = 8 days	< = 9 days	< = 10 days	< = 11 days
Unbought animal*Days*List	0.033	−0.638	−0.430	−0.336	−0.277	−0.229	−0.049	−0.087
	(0.958)	(0.516)	(0.392)	(0.372)	(0.318)	(0.305)	(0.274)	(0.267)
Close to * list	−0.291	−0.105	−0.307	−0.362	−0.288	−0.236	−0.240	−0.094
	(0.286)	(0.305)	(0.264)	(0.253)	(0.214)	(0.194)	(0.189)	(0.199)
Unbought animal*List	−0.110	−0.081	−0.082	−0.087	−0.090	−0.098	−0.109	−0.099
	(0.100)	(0.100)	(0.102)	(0.102)	(0.104)	(0.106)	(0.107)	(0.108)
List	0.199	0.204	0.200	0.210	0.230	0.224	0.243	0.223
	(0.252)	(0.253)	(0.251)	(0.250)	(0.254)	(0.254)	(0.253)	(0.253)
Non‐sensitive list A	−0.347***	−0.344***	−0.338***	−0.337***	−0.338***	−0.339***	−0.339***	−0.340***
	(0.049)	(0.048)	(0.048)	(0.048)	(0.048)	(0.048)	(0.048)	(0.049)
New * list	−0.109	−0.059	−0.016	−0.007	−0.010	−0.027	−0.045	−0.080
	(0.119)	(0.120)	(0.121)	(0.121)	(0.123)	(0.124)	(0.125)	(0.127)
Delayed * list	0.177	0.175	0.202	0.200	0.192	0.194	0.184	0.185
	(0.176)	(0.176)	(0.171)	(0.171)	(0.172)	(0.173)	(0.174)	(0.176)
FSW age * list	0.014**	0.014**	0.014**	0.014**	0.013**	0.014**	0.013**	0.014**
	(0.006)	(0.006)	(0.006)	(0.006)	(0.006)	(0.006)	(0.006)	(0.006)
Risk aversion * list	−0.046	−0.059	−0.041	−0.039	−0.042	−0.046	−0.047	−0.049
	(0.067)	(0.067)	(0.066)	(0.066)	(0.067)	(0.066)	(0.067)	(0.067)
Constant	2.077***	2.074***	2.061***	2.061***	2.034***	2.041***	2.037***	2.057***
	(0.168)	(0.169)	(0.168)	(0.168)	(0.164)	(0.165)	(0.164)	(0.164)
Observations	688	688	688	688	688	688	688	688
*R* ^2^	0.246	0.246	0.250	0.250	0.249	0.247	0.244	0.242
Effect of Tabaski on celebrators								
Linear combination	−0.258	−0.742*	−0.738**	−0.698**	−0.565**	−0.465*	−0.289	−0.18
*p*‐value	0.78	0.082	0.015	0.015	0.025	0.065	0.181	0.367
Double list experiment	Yes	Yes	Yes	Yes	Yes	Yes	Yes	Yes
Key controls	Yes	Yes	Yes	Yes	Yes	Yes	Yes	Yes
*T* ^*act*^ < 90 only	Yes	Yes	Yes	Yes	Yes	Yes	Yes	Yes
Number of FSWs	344	344	344	344	344	344	344	344

*Note:* Robust standard errors in parentheses. Specification 1 with sub‐groups (Equation (7)) with the last sex act within *T* ^*D*^ days of Tabaski defining ‘close to’ Tabaski interacted with the sub‐group of those ‘who intend to but have not yet bought an animal’ equaling 1 and those who have already bought an animal, those with no intention of equaling 0. Tabaski non‐celebrators are excluded making this a within‐Tabaski celebrators comparison. The top row is the parameter of interest, *β*_7_. Linear combination is the effect of being ‘close to’ Tabaski for celebrators. Columns from left to right are separate regressions. Data of double list experiment with FSW level clustered standard errors. The sample is limited to those who have sex acts within the last 90 days and regressions include the key controls of FSW age, new FSW to the survey, delayed interview and risk aversion. Covariates without list treatment are included but not reported for brevity. There are no sex acts within 3 days for both sub‐groups. Beyond *T* ^*D*^ < = 13 + *days* the key parameter estimates remain similar and statistically non‐significantly different from zero.

***p < 0.01, **p < 0.05, *p < 0.1.

The list experiment method is an inherently noisy method of eliciting condom use, and once we begin to perform subgroup analyses, we stretch these data, possibly beyond their useful limit. Another way to measure the effect of having not yet purchased an animal yet on condom use is to not interact *T* ^*D*^ with our subgroups but to compare across all sex acts regardless of proximity to Tabaski as per specification 3, Equation (9). Table 7 contains the results of these models. We find a condom use prevalence difference for those yet to purchase an animal of between 3.4 and 7.6 pp. However, this includes many whose last sex act was far from the influence of Tabaski, so in model 3, we estimate on a sub‐sample of FSWs whose last sex act was within 1 week of the interview, that is, *T* ^*act*^ ≤ 7. We find a statistically significant decrease in condom use prevalence of 23.4 pp in this version.

**TABLE 7:** Effect of ‘Those still to purchase an animal’ on Condom Use.

Variables	(1)	(2)	(3)
Unbought animal * list	−0.039	−0.077	−0.238**
	(0.088)	(0.089)	(0.115)
Sensitive list	0.630***	0.375*	0.541*
	(0.060)	(0.221)	(0.321)
List A	−0.339***	−0.345***	−0.365***
	(0.044)	(0.044)	(0.057)
*T^D^* continuous variable * list		0.001	0.005
		(0.002)	(0.007)
New * list		−0.123	−0.143
		(0.101)	(0.144)
FSW age * list		0.008	0.006
		(0.005)	(0.006)
Risk aversion * list		−0.033	−0.087
		(0.060)	(0.077)
Constant	2.038***	2.116***	2.148***
	(0.049)	(0.157)	(0.244)
Observations	810	808	528
*R* ^2^	0.226	0.237	0.229
Double list experiment	Yes	Yes	Yes
*T* ^*D*^ as control	No	Yes	Yes
Key controls	No	Yes	Yes
*T* ^*act*^ < 7	No	No	Yes
Number of FSWs	405	404	264

*Note*: Robust standard errors in parentheses. Regression specification 3 (Equation (9)) using the unbought animal as the defining variable of Tabaski economic pressure. The variable is defined as those ‘who intend to but have not yet bought an animal’ equaling 1 and those who have already bought an animal, those with no intention of and those not celebrating Tabaski equaling 0. The top row is the parameter of interest, *β*_3_. Data of double list experiment with FSW level clustered standard errors. All models include the key controls of FSW age, if an FSW was new to the survey and risk aversion, and interactions with sensitive list treatment plus days between last sex act and Tabaski. Delayed as a control is excluded because it is time dependent and captured in the inclusion of *T* ^*D*^. Covariates without list treatment are included but not reported for brevity. For all models the sample is limited to those who have sex acts within the last 90 days, model 3 includes those with sex acts within 7 days only. The magnitude and statistical significance of *β*_3_ in model 3 is robust for *T* ^*act*^ values less than 7, 6, 5 and 4.

***p < 0.01, **p < 0.05, *p < 0.1.

These results on economic exposure to Tabaski are based on subgroup analyses in which the usual caveats apply. Namely, we acknowledge there is some self‐selection into celebrating Tabaski. Variables used to define Tabaski celebrators or animal purchases may be correlated with other characteristics that drive any differences between subgroups, and this means we are careful to apply a causal interpretation to the sub‐group results.^26^ That said, social and religious pressures mean 83% choose to celebrate, with only 11% excluding themselves for financial reasons and since Tabaski revolves around animal sacrifice, those not purchasing an animal only do so if there are alternatives available. In addition, the results are generally clear‐cut and consistent with intuition about the FSWs who are likely to have been acutely exposed to the economic pressures of Tabaski.

#### 5.2.2 Coping strategies and relative poverty

To show whether having a relatively greater ability to cope reduces risky behavior, we estimated the difference in condom use as per specification 3, Equation (9). We use variables to indicate asset‐poor, expense‐poor and those with available savings^27^ in models 1, 3 and 5 (Table 8). We find small point estimates in the direction we expect, that is, poorer FSWs and those without savings are less likely to use condoms. However, counter‐intuitively, when we interact the coping strategy term with our acute Tabaski exposure variable, models 2, 4 and 6, we see all the signs flip, implying the poorer are more likely to use condoms if they are more exposed to Tabaski or those with savings less likely to use a condom. The counter‐intuitive signs persist when we change the savings indicator to include only those with enough savings to cover their full expected Tabaski costs.

**TABLE 8:** Effect of coping strategies on condom use.

Variables	(1)	(2)	(3)	(4)	(5)	(6)
Available savings*List					0.008	0.069
					(0.095)	(0.118)
Savings*Unbought animal*List						−0.087
						(0.144)
Expense poor*List			−0.022	−0.149		
			(0.088)	(0.112)		
Expense poor*Unbought animal*List				0.235*		
				(0.139)		
Asset poor*List	−0.009	−0.054				
	(0.090)	(0.110)				
Asset poor*Unbought animal*List		0.046				
		(0.143)				
Senitive list	0.317	0.398*	0.314	0.462**	0.310	0.356
	(0.215)	(0.223)	(0.214)	(0.222)	(0.219)	(0.226)
List A	−0.346***	−0.346***	−0.345***	−0.342***	−0.346***	−0.346***
	(0.043)	(0.044)	(0.043)	(0.044)	(0.043)	(0.044)
Sex days*List	0.001	0.001	0.001	0.001	0.001	0.001
	(0.002)	(0.002)	(0.002)	(0.002)	(0.002)	(0.002)
New*List	−0.105	−0.120	−0.103	−0.121	−0.105	−0.125
	(0.100)	(0.102)	(0.101)	(0.102)	(0.101)	(0.101)
FSW age*List	0.009*	0.008	0.009*	0.009*	0.009*	0.008
	(0.005)	(0.005)	(0.005)	(0.005)	(0.005)	(0.005)
Risk aversion*List	−0.026	−0.033	−0.025	−0.036	−0.026	−0.031
	(0.059)	(0.060)	(0.059)	(0.060)	(0.058)	(0.060)
Constant	2.260***	2.177***	2.212***	2.114***	2.137***	2.057***
	(0.152)	(0.156)	(0.152)	(0.157)	(0.152)	(0.158)
Observations	822	808	822	808	822	808
*R* ^2^	0.248	0.249	0.235	0.241	0.242	0.244
Double list experiment	Yes	Yes	Yes	Yes	Yes	Yes
*T^D^* as control	Yes	Yes	Yes	Yes	Yes	Yes
Key controls	Yes	Yes	Yes	Yes	Yes	Yes
*T* ^*act*^ < 90	Yes	Yes	Yes	Yes	Yes	Yes
Number of FSWs	411	404	411	404	411	404

*Note*: Robust standard errors in parentheses. Regression specification 3 with sub‐groups (Equation (9)) using three coping indicator variables as differentiating variables, asset poor ‐ those below median wealth index level, expense poor ‐ those below median 30 days expenses and available savings ‐ those with available savings tomorrow. Columns 1, 3 and 5 indicate association of these with condom use over the whole period. Columns 2, 4 and 6 include an interaction term with our time‐invariant Tabaski exposure variable, those ‘who intend to but have not yet bought an animal’. Data of double list experiment with FSW level clustered standard errors. All models include the key controls of FSW age, if an FSW was new to the survey and risk aversion, and interactions with list treatment plus days between last sex act and Tabaski. Delayed interviews excluded, because it is an entirely time dependent and captured in the inclusion of *T* ^*D*^. Covariates without list treatment are included but not reported for brevity. For all models the sample is limited to those who have sex acts within the last 90 days. These results are not robust to limiting the sample by *T* ^*act*^ < 7.

***p < 0.01, **p < 0.05, *p < 0.1.

Whilst these results do not give us solid evidence, we learn that the influence of wealth and coping depth is not clear cut, and policies would have to be carefully thought through and evaluated to avoid unintended consequences. It could be that any economic strengthening intended to reduce the need for condomless sex might increase participation and spending, potentially having the opposite impact on the number of condomless sex acts. Further study of interventions and possible consequences is needed.

#### 5.2.3 Client type and price

Table 9 shows the results of the changing client types in relation to Tabaski as per specification 4, Equation (10). We find a higher chance of occasional clients the closer the sex act is to Tabaski. On the one hand, this finding is unsurprising as we expect FSWs to seek new clients with an expansion of supply. On the other hand, typically, condomless sex is associated more with regular clients whom FSWs are more familiar with and have more built trust (Ferguson & Morris, 2007; Robinson & Yeh, 2012), suggesting FSWs are not only increasing their risk of infection to HIV and STIs through condomless sex but that these unprotected sex acts are likely to be occasional clients they do not know or trust as well.

The expected impact of Tabaski on prices is ambiguous. Whilst the premium for unprotected sex typically raises prices, the expected supply expansion and reduced demand due to Tabaski will lower prices. Using data on the last and penultimate sex acts for each FSWs within specification 4, Equation (10), we find no evidence that prices rise or fall depending on when a sex act takes place with respect to Tabaski, see Table A9.^28^ This finding suggests that FSWs are only able to maintain their prices whilst agreeing to more condomless sex due to the pressures of Tabaski.

**TABLE 9:** Effect of Tabaski on likelihood of occasional clients.

Variables	(1)	(2)
	Client type	Client type
*T^D^*	0.002**	0.008***
	(0.001)	(0.002)
Sex act number		−0.046**
		(0.023)
FSW age	0.002	0.003
	(0.002)	(0.002)
New FSW to the survey	−0.041	0.028
	(0.045)	(0.048)
Interview was delayed	0.133*	0.110*
	(0.070)	(0.058)
Risk aversion	0.008	0.017
	(0.026)	(0.024)
Constant	0.676***	0.477***
	(0.096)	(0.110)
Observations	411	689
*R* ^2^	0.036	0.055
Key controls	Yes	Yes
Sex‐act FE	No	Yes
Number of FSWs	411	365

*Note*: Standard errors in parentheses. Regression specification 4 (Equation (10)) with dummy variable of being a regular client as dependent variable and the number days between sex act and Tabaski as the continuous shock variable. A continuous type of *T* ^*D*^. The top row contains the parameter of interest *β*_3_ where a positive parameters is interpreted as reduced chance of regular clients as sex acts move closer to Tabaski. Model 1 is cross sectional model of last sex acts only. Model 2, a pooled OLS including both last and penultimate sex acts. Controls used were FSW age, being new to the survey, if the interview was delayed, and risk aversion. The sample was limited to having both sex acts within 28 days. Model 2 errors clustered at the FSW level.

***p < 0.01, **p < 0.05, *p < 0.1.

## 6 ROBUSTNESS CHECKS

### 6.1 New respondents

**FIGURE 5:**
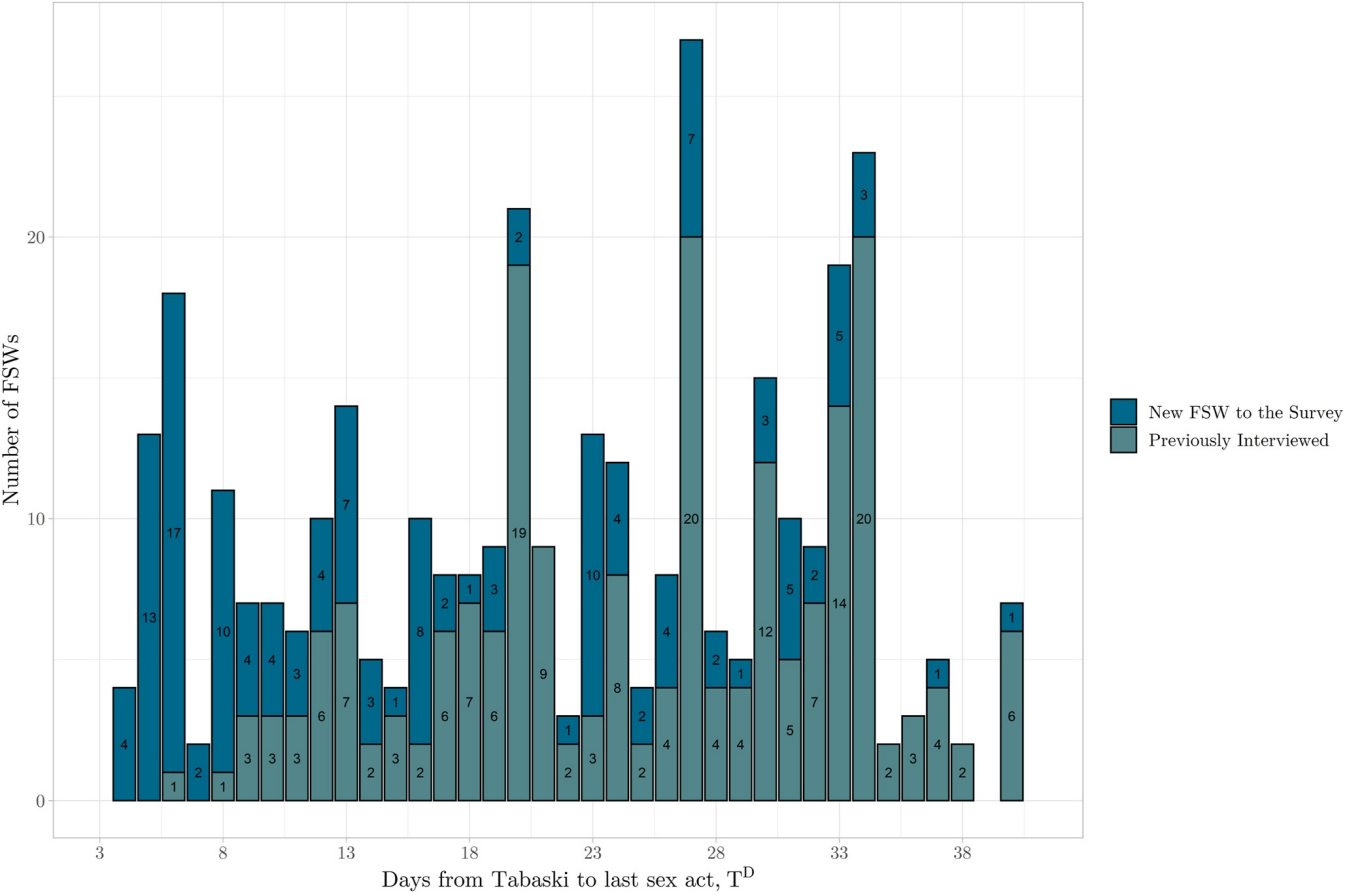
Distribution of last sex acts in relation to Tabaski, *T* ^*D*^.

**FIGURE A3:**
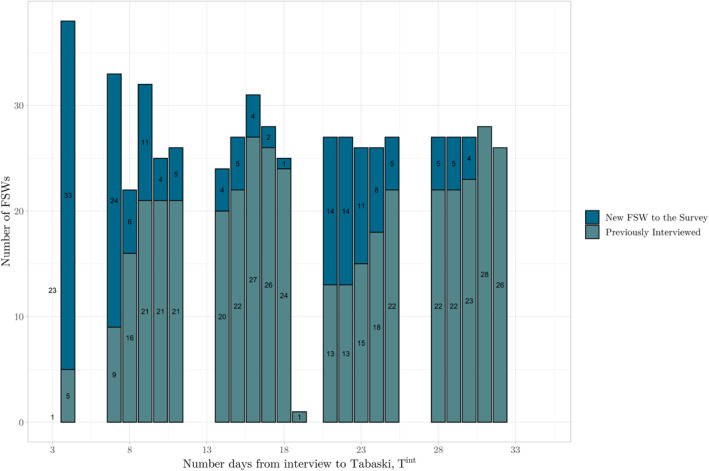
Distribution of Interview Date in relation to Tabaski, *T* ^*act*^.

As discussed in Section 4.1, new respondents were needed to maintain the cohort size, so the protocol for tracking and interviewing respondents included space for new respondents distributed across the survey. There is, however, a cluster of new respondents in the final stages of data collection, the period closer to Tabaski, see Figure A3. This was unavoidable as research teams prioritized a continuation of the panel. Figure 5 shows how our key shock variable, *T* ^*D*^, is weighted heavily toward new FSWs in the lower values of *T* ^*D*^.

If the characteristics of these new respondents are different from previously interviewed FSWs, it could threaten our identification. Data suggests the new FSWs are different in expected characteristics; that is, they are younger and are more risk‐averse (Table A5). We test if there is any relationship between condom use and if an FSW is new to the survey. Since there is a cluster of new FSWs in the survey period proximate to Tabaski, we exclude those sex acts within 14 days^29^ (the area where we see an effect of Tabaski). If new FSWs drive our results, we would expect a difference in condom prevalence between new and previously interviewed FSWs in this model. Table A6, shows no difference in condom use between being new to the survey and having been part of a previous wave.

In a further check, we examine the effect of Tabaski amongst new FSWs only using specification 1, Equation (6). Table 10 presents the results for this sub‐sample of FSWs. The effect over time found in our main results persists within new FSWs, indicating this sub‐sample does not solely drive our findings.

**TABLE 10:** Effect of Last Sex Act being ‘close to’ Tabaski on Condom Use Prevalence for New female sex workers (FSWs) only.

Variables	(1)	(2)	(3)	(4)	(5)	(6)	(7)	(8)	(9)	(10)
	*T* ^*act*^	*T* ^*act*^	*T* ^*act*^	*T* ^*act*^	*T* ^*act*^	*T* ^*act*^	*T* ^*act*^	*T* ^*act*^	*T* ^*act*^	*T* ^*act*^
	< = 4 days	< = 5 days	< = 6 days	< = 7 days	< = 8 days	< = 9 days	< = 10 days	< = 11 days	< = 12 days	< = 13 days
Close to Tabaski * list	−0.388	−0.456*	−0.432**	−0.446***	−0.329**	−0.293**	−0.259**	−0.156	−0.192*	−0.096
	(0.501)	(0.254)	(0.167)	(0.160)	(0.137)	(0.127)	(0.119)	(0.117)	(0.111)	(0.105)
Close to Tabaski	−0.274	0.141	0.217*	0.179	0.196*	0.135	0.144	0.065	0.068	0.010
	(0.258)	(0.166)	(0.112)	(0.111)	(0.103)	(0.096)	(0.092)	(0.091)	(0.082)	(0.076)
Sensitive list	0.254	0.323*	0.338*	0.349*	0.360*	0.346*	0.351*	0.313*	0.337*	0.292
	(0.184)	(0.183)	(0.181)	(0.181)	(0.186)	(0.185)	(0.186)	(0.187)	(0.189)	(0.190)
Non‐sensitive list A	−0.351***	−0.350***	−0.343***	−0.343***	−0.341***	−0.341***	−0.341***	−0.342***	−0.340***	−0.345***
	(0.043)	(0.043)	(0.043)	(0.043)	(0.043)	(0.043)	(0.043)	(0.043)	(0.043)	(0.043)
FSW age * list	0.010**	0.009**	0.009*	0.008*	0.008*	0.009*	0.009*	0.009**	0.009*	0.010**
	(0.005)	(0.005)	(0.005)	(0.005)	(0.005)	(0.005)	(0.005)	(0.005)	(0.005)	(0.005)
Risk aversion * list	−0.038	−0.044	−0.017	−0.015	−0.019	−0.022	−0.028	−0.032	−0.032	−0.036
	(0.056)	(0.056)	(0.056)	(0.056)	(0.056)	(0.056)	(0.056)	(0.056)	(0.056)	(0.056)
Constant	2.244***	2.209***	2.182***	2.187***	2.160***	2.182***	2.169***	2.200***	2.195***	2.226***
	(0.132)	(0.133)	(0.133)	(0.134)	(0.134)	(0.133)	(0.133)	(0.132)	(0.133)	(0.133)
Observations	824	824	824	824	824	824	824	824	824	824
*R* ^2^	0.234	0.234	0.236	0.237	0.234	0.234	0.233	0.231	0.232	0.231
Double list experiment	Yes	Yes	Yes	Yes	Yes	Yes	Yes	Yes	Yes	Yes
Key controls	Yes	Yes	Yes	Yes	Yes	Yes	Yes	Yes	Yes	Yes
*T* ^*act*^ < 90 only	Yes	Yes	Yes	Yes	Yes	Yes	Yes	Yes	Yes	Yes
Number of FSWs	157	157	157	157	157	157	157	157	157	157
FSWs in the ’close to’ group	4	17	35	37	48	55	62	68	78	92

*Note*: Robust standard errors in parentheses. Specification 1 (Equation (6)) using the sub‐sample of new FSWs only with the last sex act within *T* ^*D*^ days of Tabaski defining ‘close to’ Tabaski. The top row is the parameter of interest, *β*_3_. Each column is a separate regression. Data of double list experiment with FSW level clustered standard errors. The sample is limited to those who have sex acts within the last 90 days and regressions include the key controls of FSW age, new FSW to the survey, delayed interview and risk aversion. Covariates without list treatment are included but not reported for brevity. There are no sex acts within 3 days of *T* ^*D*^ < = 13 + *days* the key parameter estimates remain similar and statistically non‐significantly different from zero.

***p < 0.01, **p < 0.05, *p < 0.1.

### 6.2 Organisation

Those who lack organisational skills or have low availability because of jobs or childcare will be interviewed later in the survey period and, thus, more likely to appear in our ‘close to’ Tabaski group. Having a delayed interview could have confounding effects on condom use. From scheduling information gathered from interviewers, we determined if an interview was performed the week after it was scheduled. 30% were performed in the assigned week, 22% were performed a week before it was scheduled,^30^ and 10% were delayed. The remaining 39% were not applicable, that is, spaces for new FSWs or were not matched between the interview data and the scheduling sheets due to human data input errors.^31^ Those who took part in delayed interviews have a higher household dependency ratio, indicating they may be in busier households (Table A5), but no significant difference in condom use (Table A7). Despite not finding a link between delayed interviews and condom use, we included this as a control.

There are limitations to using an indicator for those ‘delayed’ as a control since we do not have information on scheduling for all interviews; it might not adequately capture the organisation levels of FSWs. We, therefore, test if the key confounding variable related to ‘delayed’ interviews is related to our treatment variable but find no relation, see Table 1 in the supplementary materials. In addition, as a robustness check, we perform the primary analysis using only FSWs that attended their scheduled interviews on time, supporting our main conclusions and changing our key controls to household dependency ratio, the variable strongly correlated with *delayed* interviews.^32^ Our results are robust to all of these tests and checks.

### 6.3 Weekend effect

One factor that could explain our results is the effect of the weekend. We ran the time‐invariant condom use model, specification 3, with an indicator for sex acts that took place at the weekend.^33^ Because the peak of our effect falls around the weekend before Tabaski, we exclude the sex acts within 7 days of Tabaski. Table 5 in the supplementary materials shows no evidence of weekends leading to lower condom use. Our main results are also robust to include a weekend dummy as a covariate.^34^

### 6.4 Migration and changing client pool

Another reason to explain our findings is migration or differential attrition due to Tabaski. Migration of FSWs and clients out of the city, or migration in of their families, might change the likelihood of response and, therefore, the pool of FSWs or clients available for interview close to Tabakski in a way related to condom use. For example, FSWs and clients may be unable to solicit clients if their families come to stay. Broadly, migration might be affecting the entire sample such that our sample excludes those who regularly travel out of the city for more prolonged periods around Tabaski. But we know from scheduling information that only a very small portion (1%, *n* = 4)^35^ could not take part or delay an interview because of travel. The attrition rates were similar between waves one and two (Wave one was an interview period far from Tabaski and wave two close to Tabaski) and waves two and three (both proximate to Tabaski), implying no differential level of migration influencing the pool of FSWs available for the survey because of Tabaski. Table A1, column 1, in the Appendix, also shows no relationship between FSW characteristics and the interview date.

There are three reasons why we do not think migration within our sample explains our results. First, typical workers in Dakar receive only 2 days off for Tabaski, the day of the celebration and the following day. Because of this, migration into or out of Dakar typically occurs between zero and 4 days before the feast, dependent on individual circumstances. In our dataset, the closest sex act we identify is 4 days before the celebration and the closest interview 3 days before, indicating little chance of significant client or family migration. Second, we do find an increase in the likelihood of occasional clients but find no difference in condom use between regular and occasional clients, see Table 6 in the supplementary materials. In addition, the literature finds occasional clients typically are associated with greater condom use (Ferguson & Morris, 2007; Robinson & Yeh, 2012), implying the increase in occasional clients does not explain our results. Third, if FSWs found it more difficult to seek clients because of family arriving^36^ or some other Tabaski‐related reason, we would expect the time since the last sex act to be higher for those interviewed closer to the festival. For those interviewed 3 and 4 days (*T* ^int^ = 3 and *T* ^int^ = 4) before the festival, the mean *T* ^*act*^ is 8.3 and 6.2, respectively. For those interviewed in the first week of interviews (*T* ^int^ = 28 to *T* ^int^ = 32) the mean *T* ^*act*^ is between 6.4 and 14.3. Unadjusted regression finds no relationship between these *T* ^*act*^ and *T* ^int^ either.^37^

We test the change in FSW‐reported client characteristics to observe changes closer to Tabaski. Our findings show that clients are less likely to be ”as clean” or ”as good‐looking” as an FSW's typical client (equally likely to be better or worse) but that the risk of HIV, perceived wealth and age do not differ, Table A10. These findings are consistent with our prediction that the supply of sex increases, but demand falls without a significant change in the pool of clients. Even if non‐shock channels drove our results, Tabaski is still strongly associated with large reductions in condom use. To explore the potential other channels, richer data on clients and multiple sex act information before and after Tabaski for FSWs would be needed.

### 6.5 Direct questionning

We estimated our results using the answers to the direct question of if a condom was used during the last sex act, where 97% said ‘yes’, and unsurprisingly found no evidence of Tabaski influencing condom use, proving the value of the list experiment. Since we used the double list experiment for our main analysis, we also tested using each side of the list experiment, finding similar results; see Figure 1 in the supplementary materials. Our results were robust to the inclusion of *T* ^*act*^ and dependency ratio as key controls; see Figures 3 and 2 in the supplementary materials.

## 7 DISCUSSION

In this study, we assess the impact of a significant religious festival, Tabaski, on the risky sexual behaviors of FSWs. We identified a significant reduction in condom use in at least the 9 days prior to Tabaski. In the 7 days before the feast, we find up to a 49.5 pp drop in condom use. We find that those who are yet to buy an animal at the time of the interview have a condom prevalence 23.4 pp lower than those who have purchased an animal, with this effect peaking in the final 7 days before Tabaski when it is unlikely any sex acts are protected for this sub‐group. We find no difference in price consistent with the idea that any premium associated with condomless sex is cancelled out by decreased demand and increased supply of risky sex. There is also little evidence that savings or wealth protects against changes in behaviors; however, we cannot say for certain due to data and analysis limitations. We show our results are robust to several potential confounders, including days since the FSW's last sex act, those ‘new to the survey’, those with ‘delayed’ interviews, sex acts at the weekend, and a number of reasons why migration might threaten our results.

Data collection took place during the COVID pandemic of 2020 and could mean our results are a one‐off. Whilst lockdowns and restrictions had largely ceased by the time our data was collected, bars and nightclubs, a key source of clients, remained closed. Cust et al. (2021), find a reduction in clients and earnings, particularly for those who have difficulty borrowing. This is consistent with the idea that COVID is having a depressive effect on the local sex economy such that condomless sex is the only avenue left available, which, as we have seen, does not lead to an overall increase in the prices. A second consequence is that coping strategies (*A* in our conceptual framework) may have been exhausted coping with COVID such that there is little left to deal with Tabaski, so being underprepared this year is unique. However, debt and savings remained relatively stable between wave two and wave three; households in debt increased 3%, and those with savings fell 4%, but the quantity for those with savings rose 20%, although the quantity might be due to the 2020 data collection period being slightly closer to Tabaski.

The design of our analysis means our results are an internal comparison only. Considering the size of the shock (the expected cost of animals is 121% of typical monthly sex work income), it is plausible that the economic pressure runs across the entire period we collected data, meaning our comparison group is not a good approximation of an FSW's behavior the rest of the year. Should this be the case, our results are likely an underestimation. Further evidence from Treibich and Lépine (2019) using the same longitudinal dataset of FSWs shows there was no significant difference in condom use between data collection in wave one and in wave two (79.6% and 78.2%), with the former being collected at a different time of year (both by calendar and in relation to Tabaski), and the latter being collected at the similar time of year and one week prior (about Tabaski) than wave three in 2020.^38^ The consistency of findings between wave one and wave two implies no calendar or seasonal difference and no longer‐term Tabaski shock difference in condomless sex.

Our study is relevant for all FSWs in Sub‐Saharan Africa that celebrate Tabaski and, more broadly, for economic shocks with similar characteristics. The original sample was not entirely representative, given the requirement for a 50‐50 split of registered and unregistered FSWs and the observational nature of the dataset tracking the same FSWs over time. We used respondent‐driven sampling methodologies that are best practice for these populations (Magnani et al., 2005) both for the initial FSWs and replacements in subsequent waves meaning the sample naturally cannot drift too far from the underlying population. Indeed, the balance of registered to unregistered has moved from 50% in wave 1%–53%, closer to the 57% previously found (APAPS & IRESSEF, 2014).^39^ We, therefore, are confident our findings apply to the wider FSW population in Dakar.

Considering the implications more widely, Senegal's unique legal and contextual frameworks surrounding sex work make direct applications to other countries less straightforward. The inclusion of unregistered FSWs somewhat mimics FSWs in countries where sex work is illegal and where FSWs must take precautions to remain undiscovered, meaning our results do have substantial implications across the continent. A key population not captured here is those who engage in transactional sex but do not identify as sex workers. These women are also exposed to similar premiums and incentives as sex workers but are likely a much larger population than self‐identifying sex workers (Luke, 2006; Stoebenau et al., 2016; Wamoyi et al., 2019). Our results suggest further research of anticipated shocks in transactional sex populations is much needed, particularly among adolescent and young women where HIV incidence is greatest.

Our study differs from the economic shocks literature because we study an anticipated economic shock that some theories would predict would be smoothed away. The response we observe is consistent with the effects seen for unanticipated shocks, but our estimates' magnitude is much larger. However, not all economic shock studies find increases in risky behaviors or HIV and STI health outcomes (Cust et al., 2021). Aker et al. (2020) is the only other study to investigate Tabaski, albeit not in a sexual health context. It also finds that Tabaski exerts significant economic pressure on households but that their savings intervention does not help smooth consumption when a shock is anticipated.

Any policy has to be carefully designed and should focus on easing the economic pressure in the final week before Tabaski without increasing intended spending, which could lead to little effect on behaviors.^40^ Supplying animals to FSWs free of charge, a voucher system that can be redeemed for a Tabaski animal, or a cash transfer could lessen the risky behavioral responses by smoothing the spike in economic pressure from animal prices. Less costly solutions, such as financial education and savings interventions specifically targeted toward Tabaski, such as Aker et al. (2020), or similar to Jones and Gong (2021) with earmarked accounts could work with special attention given to preventing unintended consequences. From a public health point of view, these policies should be available to all FSWs or vulnerable women at risk of entering the commercial sex market. However, care should be taken to avoid policies which might increase expected Tabaski spending and inadvertently increase risky sexual behaviors.

Our study has several limitations. The list experiment is inherently noisy and inefficient with low statistical power and has limitations on the type of analysis we could perform, such as calculating risk premiums. It means we cannot draw strong conclusions around the heterogeneous effects of savings and wealth. Surveys asked FSWs to recall their last paid sex acts, which adds potential recall issues. Because we only have a single sex act per FSW our results reflect the propensity for an individual FSW, to use a condom at their last sex act only. We cannot adequately analyze the intensity of condomless sex acts using these data, so we cannot answer the more pertinent public health questions about the total number of condomless sex acts. Future research should focus on directly measuring health impacts, for example, HIV and STIs, following Tabaski or shocks with similar characteristics, plus repeated data collection before, during, and after such events through sex act diaries. There should be a focus on interactions of shocks with coping strategies to inform policies better to protect against such shocks whilst avoiding unintended consequences.

## 8 CONCLUSION

How FSWs and women vulnerable to transactional sex respond to economic hardship is vital to aid efforts to improve sexual health and reduce HIV spread in low‐ and middle‐income countries. Our paper seeks to identify if there is a behavioral response of FSWS to anticipated economic shocks similar to the effects found for unanticipated shocks. We found that anticipation and knowledge of upcoming economic shocks do not lead to adequate savings, and its magnitude meets the threshold for a catastrophic health expense. Female sex workers respond by increasing risk‐taking in sexual behaviors. We found those with sex acts within 9 days of Tabaski were less likely to use condoms, with a reduction in condom use prevalence of up to 49.5 pp (76%) compared to sex acts furthest from the festival. We show that the economic component of the festival is highly likely to be driving the observed drop in condom use and that those who are yet to buy an animal are unlikely to be using condoms at all in the six to 8 days before the festival. Tabaski has never before been documented as a cause of risky behaviors and has been shown to lead to condomless sex in a key population at high risk of HIV for at least 1 week every year. Our findings have important public health policy implications for FSWs affected by anticipated shocks with limited shock‐coping strategies. We highlight the importance of protection against anticipated and unavoidable shocks as well as unanticipated shocks.

## CONFLICT OF INTEREST STATEMENT

None.

## Supporting information

Supporting Information S1

## Data Availability

Research data are not shared.

## ENDNOTES

^1^
See The Strive group's work for detailed discussions of transactional sex and its contribution to HIV (Stoebenau et al., 2016; Wamoyi et al., 2019)

^2^
Indeed, those in agriculture make earnings from their harvest last the year (Rosenzweig & Wolpin, 1993), but in general it is more frequent to miss short and long‐run savings goals.

^3^
Some argue a ‘shock’ implies it is unexpected and ‘economic pressure’ may be more suitable. We argue that if a large, anticipated expense is unavoidable and impacts household expenditure, it is a shock. We use shock and pressure interchangeably in this paper.

^4^
Also known as ‘list randomisation’ or ‘item count’ methods.

^5^
Typically FSWs do not receive support from government orNGOs for Tabaski but they do for some other shocks such as free treatment for STIs.

^6^
Access to finance, particularly consumption finance, is difficult for vulnerable populations ‐ only 53% of our sample has a mobile banking account or a bank account, and a similar proportion were already in debt. Our measure is not nuanced enough to tell us if household debt indicates access to finance to help with shocks or if the costs of debt are straining household finances further.

^7^
Reasons for not celebrating: Non‐Muslim: 19%, No Money: 70%, COVID‐19: 3%, Other: 7%

^8^
All exchange rates calculated at 1 USD = 553.000 XOF 31st July 2020.

^9^
Many share the costs as this is a family celebration often with many others contributing.

^10^
Looking at actual earnings in the last 30 days makes the cost of animals proportionally higher. However, we are unable to disentangle possible COVID‐19 and Tabaski effects on the last 30 days' earnings, hence we compare using the *typical* reported earnings. Kavanagh et al. (2021) provide evidence of possible COVID effects on the earnings of these FSWs.

^11^
Due to both farmers investing in transporting them to areas of high demand and because of spikes in demand themselves (Apolloni et al., 2018). Excess supply doesn't necessarily mean lower prices either, as the livestock remains valuable to farms after Tabaski

^12^
A mean of 4 days when combining ‘those already bought’ with those who have and have not yet bought the animal.

^13^
Based on the average cost in 2020.

^14^
Data collection in 2017 took place at a similar time in relation to Tabaski. Unfortunately, there were no questions about the effects of Tabaski asked, meaning we have limited areas of comparison.

^15^
‘Typical’ in the survey might have been interpreted as pre‐COVID too, although we did not specify, so the difference is hard to attribute to Tabaski alone.

^16^
There is debate over the effectiveness of the list experiment in measuring sensitive behaviors. Lensvelt‐Mulders et al. (2016) perform a meta‐analysis finding it more accurate than direct face‐to‐face questioning at estimating prevalence, whereas several other studies find issues often derived from the implementation of the method, including whether it is understood by respondents (Haber et al., 2018).

^17^
Our main results are robust to using single‐sided list experiment analysis, see Figure 1 in the supplementary materials.

^18^
The measure of risk aversion is derived from the Gneezy‐Potter investment game that determines risk aversion of individuals with values of 0 to 2 (Charness & Gneezy, 2010). All results are robust to the removal of controls and are explored later in the analysis. We do not include all possible controls for the risk of over‐fitting.

^19^
We do not have enough data for each value of *T* ^*D*^ to perform this using each day as a dummy variable. We use larger blocks to give ourselves suitable statistical power to draw conclusions.

^20^
Wedo not extend our analysis here to use fixed effects because first differences in the continuous *T* ^*D*^ simply represent the difference in time between the last two sex acts of the FSW which does not reflect changes in Tabaski pressure.

^21^
We run but do not report the results using the incremental changes in *T* ^*D*^ similar to specifications 1 and 2, Equations (6) and (8) for these outcomes too.

^22^
These results are robust to the inclusion of *T* ^*act*^ as an additional control, see Figure 4 in the supplementary materials. It is also robust to incrementally reducing the sample from *T* ^*act*^ ≤ 90 to *T* ^*act*^ ≤ 1. In other words even including only FSWs who had sex within the last 24 h the results hold. The strongest effect is found when *T* ^*act*^ is limited to around 1 week, implying our results are not driven by the comparison group containing a high mean of *T* ^*act*^, see Figure 5 in the supplementary materials

^23^
This is calculated using the value of *β*_3_ when *T* ^*D*^ = 7, 0.431, from specification 1, Table 3, and the condom prevalence for the comparison group taken from specification 1 without controls, Table A4, 0.658. The controlled specification with the list experiment does not give an accurate figure for the prevalence of the comparison group.

^24^
The models without controls are available on request.

^25^
Versions of both without controls support our findings and are available on request.

^26^
Tabaski celebrators are more likely to be Muslim, in the poorest wealth quintile, be less risk‐averse and be a new FSW to the survey. Those yet to buy an animal are more likely to be less risk‐averse and typically earn more. See Table 4 in Supplementary Materials.

^27^
Asset‐poor and expense‐poor are dummy variables equaling 1 when an FSW is below the median level of our asset wealth index or the median level of 30‐day expenses across the whole sample. Available savings is a dummy variable equaling 1 when an FSW has a non‐zero level of savings.

^28^
Because we cannot attach condom use to the individual FSWs and given the impossibility to use a variable measured with the list experiment as a right‐hand‐side variable we cannot investigate how the risk premium changes with Tabaski. We also estimate versions using the definition of ‘close to’ Tabaski in specifications 1 & 2, Equations (6) and (8) again finding no effect of Tabaski on prices.

^29^
Results are consistent reducing the exclusion period down to *T* ^*D*^ < five

^30^
Enumerators were instructed to move to the next week of their list if they had exhausted their options for that week thus continuing with the randomisation so these interviews do not pose a problem to our identification.

^31^
These were aids for the interviewers and were not originally intended for analysis.

^32^
Results available in Tables 2 and 3 and Figure 2 in supplementary materials.

^33^
We test with three definitions: 1) Saturday & Sunday, 2) Friday & Saturday, 3) Friday, Saturday & Sunday.

^34^
Results for the weekend indicator included as a covariate and other definitions of weekend are available on request.

^35^
This information is drawn from the scheduling information recorded by interviewers and was not designed for analysis. The information was incomplete

^36^
It is worth noting that our interviews took place in private and at a location that does not indicate the respondents' occupation.

^37^
Available on request.

^38^
Survey 2015: 18^*th*^ May ‐ 2^*nd*^ July. Tabaski 2015: Around 23^*rd*^ ‐ 27^*th*^ September. Survey 2017: 7^*th*^ August ‐ 26^*th*^ August. Tabaski 2017: 2^*nd*^ September. Survey 2020: June 29^*th*^ ‐ July 28^*th*^ Tabaski 2020: 31^*th*^ July

^39^
There have been no substantial changes in the registration policy or local attitudes to FSWs, meaning we believe the true proportion of registered has not differed much from this figure.

^40^
An important aspect of Tabaski is sharing of the sacrificial animal with the community, and so with additional wealth FSWs may set their sights higher. There is some evidence that richer FSWs spend more than poorer. For every additional 1 CFAF of typical monthly earnings, an FSW expects to spend 0.16 CFAF more in total on Tabaski.

## APPENDIX A Tables and Figures

**TABLE A1:** Determinants of *T* ^int^ and *T* ^*act*^.

	(1)	(2)	(3)
Variables	T^int^	T^*act*^	T^*act*^
Registered	−0.562	1.054	1.979
	(0.533)	(0.594)	(0.371)
FSW age	−0.003	0.116	−0.050
	(0.956)	(0.340)	(0.750)
Gneezy‐Potter risk preference/2	−0.437	−0.288	−0.341
	(0.421)	(0.809)	(0.790)
Time preference	0.481	−3.907*	−3.171
	(0.637)	(0.082)	(0.178)
Number of children	−0.002	−0.385	−0.465
	(0.988)	(0.255)	(0.178)
Dependency ratio	0.029	1.050**	1.011**
	(0.886)	(0.020)	(0.030)
New FSW to survey	−8.806***	−1.209	−1.295
	(0.000)	(0.553)	(0.547)
Intensity ‐ typical number of clients in 7 days	−0.038	−0.136	−0.177
	(0.619)	(0.419)	(0.339)
Marital status: Married	−2.877	17.612	17.042
	(0.622)	(0.170)	(0.191)
Marital status: Divorced or separated	−0.871	2.254	2.600
	(0.432)	(0.354)	(0.312)
Marital status: Widowed	−1.734	8.174*	8.280*
	(0.366)	(0.053)	(0.061)
Logged typical earnings (all sources)	−0.637	−2.169	−2.368
	(0.324)	(0.126)	(0.117)
Both parents are alive	−0.742	3.420	3.718
	(0.468)	(0.128)	(0.110)
Both parents are dead	−0.323	−1.997	−1.877
	(0.760)	(0.391)	(0.437)
Constant	30.599***	31.640*	30.964*
	(0.000)	(0.068)	(0.098)
Observations	409	409	398
*R* ^2^	0.281	0.097	0.125
FSW Covariates	Yes	Yes	Yes
Wealth covariates	Yes	Yes	Yes
Client/sex‐act covariates	No	No	Yes
*T* ^*act*^ < 90 only	Yes	Yes	Yes
Number of FSWs	409	409	398

*Note*: *p*‐values in parentheses. Model 1 is *T* ^int^ regressed on FSW characteristics. Last sex characteristics are not included as these are likely influenced somewhat by Tabaski. Model 2 is *T* ^*act*^ regressed on FSW characteristics, model 3 includes last sex characteristics (unreported). All unreported variables are not statistically significant at 1% or 5% levels. Marital status reference category ‐ never married. Unreported wealth quintiles, reference category ‐ middle quintile. Unreported education categories, reference category ‐ no education. Other unreported last sex characteristics: age of client, regular or occasional, client risk of HIV, if FSW or client consumed alcohol, negotiation took place, if the sex act took place in public, sex act duration, if fellatio or anal sex took place, if the client was rich, if the FSW stayed the night. All are self‐reported by the FSW. The sample is limited to those who have sex acts within the last 90 days. Gneezy‐Potter Risk preference is an investment game to determine the risk aversion of individuals with values of 0 to 2 (Charness and Gneezy, 2010). Female sex worker household dependency ratio is the ratio of children and under 65's to adults in the FSWs household. Time preference is a percentage of those who prefer money today instead of twice as much in 1 weeks time.

****p* < 0.01, ***p* < 0.05, **p* < 0.1.

**TABLE A2:** Effect of days since last sex Act on condom use prevalence.

	(1)	(2)	(3)	(4)	(5)	(6)
Variables	*T* ^*act*^ continuous	*T* ^*act*^ continuous	Dummy ‐ *T* ^*act*^ < = 3	Dummy ‐ *T* ^*act*^ < = 3	Dummy ‐ *T* ^*act*^ < = 7	Dummy ‐ *T* ^*act*^ < = 7
*T* ^*act*^ * List	0.002	0.002	0.032	0.007	0.061	0.042
	(0.002)	(0.002)	(0.061)	(0.063)	(0.063)	(0.064)
Sensitive list	0.594***	0.645***	0.652***	0.688***	0.626***	0.666***
	(0.052)	(0.072)	(0.060)	(0.069)	(0.068)	(0.075)
*T* ^*act*^	−0.003**	−0.003**	−0.069	−0.050	−0.013	0.004
	(0.001)	(0.001)	(0.087)	(0.090)	(0.088)	(0.090)
Non‐sensitive list A	−0.338***	−0.336***	−0.339***	−0.338***	−0.339***	−0.337***
	(0.043)	(0.043)	(0.043)	(0.043)	(0.043)	(0.043)
Intensity * list		−0.007		−0.007		−0.008
		(0.006)		(0.006)		(0.006)
Intensity		0.008*		0.009*		0.009*
		(0.005)		(0.005)		(0.005)
Constant	2.140***	2.080***	2.087***	2.038***	2.063***	2.016***
	(0.043)	(0.055)	(0.048)	(0.055)	(0.054)	(0.059)
Observations	824	824	824	824	824	824
*R* ^2^	0.225	0.227	0.222	0.225	0.222	0.225
Intensity control	No	Yes	No	Yes	No	Yes
*T* ^*act*^ < 90 only	Yes	Yes	Yes	Yes	Yes	Yes
Number of FSWs	412	412	412	412	412	412

*Note*: Robust standard errors in parentheses. Model 1 & 2 uses *T* ^*act*^ as a continuous variable, model 3 & 4 a dummy variable equal 1 if *T* ^*act*^ < = 3 and model 5 & 6 equal 1 if *T* ^*act*^ < = 7. Models 2, 4 & 6 include the intensity variable ‐ typical number of clients in 7 days leaving any remaining relationship to recall bias only. The sample is limited to those who have sex acts within the last 90 days. The cut off for the dummy was repeated up to *T* ^*act*^ = 21 at which point there does become a small and statistically significant effect within our sample.

****p* < 0.01, ***p* < 0.05, **p* < 0.1.

**TABLE A3:** Determinants of *T* ^*D*^.

	(1)	(2)	(3)	(4)
Variables	*T* ^*D*^ continuous	*T* ^*D*^ < = 7	*T* ^*D*^ < = 10	*T* ^*D*^ < = 14
Registered	−0.410	−0.039	0.003	−0.007
	(0.847)	(0.194)	(0.933)	(0.874)
FSW Age	0.102	−0.001	−0.003	−0.003
	(0.444)	(0.449)	(0.206)	(0.240)
Gneezy‐Potter risk preference/2	−0.423	0.033*	0.012	0.000
	(0.746)	(0.073)	(0.598)	(0.987)
Time preference	−3.460	−0.010	−0.005	0.033
	(0.158)	(0.781)	(0.910)	(0.508)
Number of children	−0.269	0.005	−0.000	0.003
	(0.471)	(0.371)	(0.989)	(0.683)
Dependency ratio	0.814	−0.002	−0.004	−0.010
	(0.117)	(0.773)	(0.655)	(0.339)
New FSW to survey	−10.129***	0.210***	0.280***	0.321***
	(0.000)	(0.000)	(0.000)	(0.000)
Intensity ‐ typical number of clients in 7 days	−0.203	0.003	0.003	0.003
	(0.272)	(0.236)	(0.272)	(0.470)
Wealth index continuous	1.208	−0.006	−0.005	−0.016
	(0.195)	(0.673)	(0.750)	(0.396)
Logged typical earnings (all sources)	−2.365	0.014	0.017	0.016
	(0.123)	(0.510)	(0.510)	(0.596)
Both parents are alive	2.706	0.002	0.034	0.038
	(0.270)	(0.955)	(0.420)	(0.452)
Both parents are dead	−1.793	0.017	0.015	0.068
	(0.482)	(0.637)	(0.730)	(0.191)
Constant	58.479***	−0.143	−0.076	−0.002
	(0.002)	(0.582)	(0.811)	(0.997)
Observations	408	409	409	409
*R* ^2^	0.135	0.172	0.201	0.179
FSW Covariates	Yes	Yes	Yes	Yes
Coping strategies	Yes	Yes	Yes	Yes
*T* ^*act*^ < 90 only	Yes	Yes	Yes	Yes
Number of FSWs	408	409	409	409

*Note*: *p*‐value in parentheses. Model 1 regresses a binary variable equal 1 if *T* ^*D*^ < = 7, model 2 if *T* ^*D*^ < = 10, and model 3 *T* ^*D*^ < = 14. Last sex characteristics are not included as these are likely influenced somewhat by Tabaski. All unreported variables are not statistically significant at 1% or 5% levels. Unreported marital status reference category ‐ never married. Unreported education categories, reference category ‐ no education. The sample is limited to those who have sex acts within the last 90 days. Gneezy‐Potter Risk preference is an investment game to determine the risk aversion of individuals with values of 0 to 2 (Charness and Gneezy, 2010). Female sex worker household dependency ratio is the ratio of children and under 65's to adults in the FSWs household. Time preference is a percentage of those who prefer money today instead of twice as much in 1 weeks time.

****p* < 0.01, ***p* < 0.05, **p* < 0.1.

**TABLE A4:** Effect of Last Sex Act being ‘close to’ Tabaski on Condom Use Prevalence without Controls.

Variables	(1)	(2)	(3)	(4)	(5)	(6)	(7)	(8)
	*T^D^*	*T* ^*D*^	*T* ^*D*^	*T* ^*D*^	*T* ^*D*^	*T* ^*D*^	*T* ^*D*^	*T* ^*D*^
	< = 4 days	< = 5 days	< = 6 days	< = 7 days	< = 8 days	< = 9 days	< = 10 days	< = 11 days
Close to Tabaski * list	−0.465	−0.522**	−0.486***	−0.499***	−0.391***	−0.348***	−0.314***	−0.216*
	(0.565)	(0.259)	(0.165)	(0.157)	(0.133)	(0.125)	(0.118)	(0.117)
Close to Tabaski	−0.265	0.161	0.212*	0.176	0.192*	0.135	0.147	0.073
	(0.254)	(0.167)	(0.111)	(0.109)	(0.102)	(0.095)	(0.092)	(0.090)
Sensitive list	0.621***	0.638***	0.658***	0.661***	0.662***	0.663***	0.664***	0.652***
	(0.043)	(0.044)	(0.044)	(0.045)	(0.046)	(0.046)	(0.047)	(0.047)
Non‐sensitive list A	−0.344***	−0.343***	−0.336***	−0.336***	−0.334***	−0.334***	−0.334***	−0.332***
	(0.043)	(0.043)	(0.043)	(0.043)	(0.043)	(0.043)	(0.043)	(0.043)
Constant	2.109***	2.099***	2.085***	2.087***	2.079***	2.083***	2.079***	2.089***
	(0.038)	(0.038)	(0.038)	(0.038)	(0.038)	(0.039)	(0.039)	(0.039)
Observations	824	824	824	824	824	824	824	824
*R* ^2^	0.226	0.227	0.230	0.231	0.228	0.228	0.227	0.224
Double list experiment	Yes	Yes	Yes	Yes	Yes	Yes	Yes	Yes
Key controls	Yes	Yes	Yes	Yes	Yes	Yes	Yes	Yes
*T* ^*act*^ < 90 only	Yes	Yes	Yes	Yes	Yes	Yes	Yes	Yes
Number of FSWs	412	412	412	412	412	412	412	412
FSWs in ’close to’ group	4	17	35	37	48	55	62	68

*Note*: Robust standard errors in parentheses. Specification 1 (Equation (6)) with the last sex act within *T* ^*D*^ days of Tabaski defining ‘close to Tabaski’. The top row is the parameter of interest, *β*_3_. Each column is a separate regression. Data of double list experiment with FSW level clustered standard errors. The sample is limited to those who have sex acts within the last 90 days and regressions does not key controls. There are no sex acts within 3 days of Tabaski and *T* ^*D*^ < = 11 + *days* the key parameter estimates remain similar and statistically non‐significantly different from zero, see Figure A2.

****p* < 0.01, ***p* < 0.05, **p* < 0.1.

**TABLE A5:** Determinants of new female sex workers (FSWs) and delayed interviews.

	(1)	(2)
Variables	New FSWs	Delayed interviews
Registered	0.059	1.052
	(0.248)	(0.595)
FSW Age	−0.016***	0.104
	(0.000)	(0.392)
Gneezy‐Potter risk preference/2	0.110***	−0.239
	(0.000)	(0.841)
Time preference	−0.030	−3.879*
	(0.612)	(0.084)
Number of children	−0.012	−0.380
	(0.176)	(0.261)
Dependency ratio	−0.009	1.015**
	(0.466)	(0.024)
Intensity ‐ typical number of clients in 7 days	0.001	−0.158
	(0.838)	(0.347)
Marital status: Married	0.359	17.465
	(0.282)	(0.174)
Marital status: Divorced or separated	−0.071	2.129
	(0.259)	(0.381)
Marital status: Widowed	−0.057	7.946*
	(0.601)	(0.060)
New FSW to survey		−1.828
		(0.351)
Logged typical earnings (all sources)	0.024	−1.932
	(0.514)	(0.169)
Both parents are alive	−0.046	3.658
	(0.427)	(0.102)
Both parents are dead	0.051	−1.694
	(0.397)	(0.464)
Constant	0.757*	30.000*
	(0.092)	(0.083)
Observations	409	409
*R* ^2^	0.176	0.094
FSW Covariates	Yes	Yes
Wealth covariates	Yes	Yes
Client/sex‐act covariates	No	No
*T* ^*act*^ < 90 only	Yes	Yes
Number of FSWs	409	409

*Note:* *p*‐value in parentheses. Model 1 is ‘new FSWs to the survey’ regressed on FSW characteristics. Last sex characteristics are not included as these are likely influenced somewhat by Tabaski. Model 2 is ‘delayed interviews’ regressed on FSW characteristics. All unreported variables are not statistically significant at 1% or 5% levels. Marital status reference category ‐ never married. Unreported wealth quintiles, reference category ‐ middle quintile. Unreported those with a second job. Unreported education categories, reference category ‐ no education. The sample is limited to those who have sex acts within the last 90 days. Gneezy‐Potter Risk preference is an investment game to determine the risk aversion of individuals with values of 0 to 2 (Charness and Gneezy, 2010). Female sex worker household dependency ratio is the ratio of children and under 65's to adults in the FSWs household. Time preference is a percentage of those who prefer money today instead of twice as much in 1 weeks time.

***p < 0.01, **p < 0.05, *p < 0.1.

**TABLE A6:** Condom use prevalence of new and previously interviewed female sex workers (FSWs).

Variables	(1)	(2)	(3)	(4)
New FSW*Sensitive list	−0.103	−0.043	−0.044	−0.011
	(0.110)	(0.118)	(0.156)	(0.174)
New FSW to the survey	0.100	0.076	0.080	0.080
	(0.080)	(0.083)	(0.107)	(0.113)
Sensitive list	0.698***	0.304	0.670***	0.558*
	(0.052)	(0.214)	(0.078)	(0.311)
List A	−0.361***	−0.366***	−0.426***	−0.430***
	(0.046)	(0.045)	(0.068)	(0.069)
FSW age * sensitive list		0.010*		0.004
		(0.005)		(0.008)
Risk aversion * sensitive list		−0.003		−0.066
		(0.060)		(0.091)
Constant	2.050***	2.241***	2.150***	2.122***
	(0.041)	(0.144)	(0.061)	(0.213)
Observations	734	734	372	372
*R* ^2^	0.268	0.272	0.266	0.268
Double list experiment	Yes	Yes	Yes	Yes
Key controls	No	Yes	No	Yes
*T^D^* > = 10	Yes	Yes	Yes	Yes
*T* ^*act*^ < = 7	No	No	Yes	Yes
Number of FSWs	345	345	177	177

*Note*: Robust standard errors in parentheses. Similar to specification 3 (Equation (9)) looking at the effect of New FSWs on condom use. The top row is the parameter of interest *β*_3_. Data of double list experiment with FSW level clustered standard errors. All models are limited to sex acts more than 10 days from Tabaski. Models 1 & 3 are without controls. Models 2 & 4 includes controls ‐ FSW age, risk aversion. Models 3 & 4 include only sex acts within 7 days of the interview. Covariates without list treatment are included but not reported for brevity.

****p* < 0.01, ***p* < 0.05, **p* < 0.1.

**TABLE A7:** Condom use prevalence of female sex workers (FSWs) with delayed interviews.

Variables	(1)	(2)	(3)	(4)
Delayed*Sensitive list	0.027	0.051	0.250	0.259
	(0.137)	(0.139)	(0.220)	(0.228)
Sensitive list	0.672***	0.267	0.641***	0.513*
	(0.048)	(0.197)	(0.071)	(0.276)
List A	−0.358***	−0.365***	−0.424***	−0.430***
	(0.046)	(0.045)	(0.067)	(0.069)
FSW age * sensitive list		0.010**		0.005
		(0.005)		(0.007)
Risk aversion * sensitive list		−0.005		−0.066
		(0.060)		(0.088)
Constant	2.076***	2.303***	2.178***	2.199***
	(0.041)	(0.140)	(0.059)	(0.205)
Observations	734	734	372	372
*R* ^2^	0.266	0.271	0.266	0.268
Double list experiment	Yes	Yes	Yes	Yes
Key controls	No	Yes	No	Yes
*T^D^* > = 10	Yes	Yes	Yes	Yes
*T* ^*act*^ < = 7	No	No	Yes	Yes
Number of FSWs	345	345	177	177

*Note*: Robust standard errors in parentheses. Similar to specification 3 (Equation (9)) looking at the effect of FSWs with delayed interviews on condom use. The top row is the parameter of interest *β*_3_. Data of double list experiment with FSW level clustered standard errors. All models are limited to sex acts more than 10 days from Tabaski. Models 1 & 3 are without controls. Models 2 & 4 includes controls ‐ FSW age, risk aversion. Models 3 & 4 include only sex acts within 7 days of the interview. Covariates without list treatment are included but not reported for brevity.

****p* < 0.01, ***p* < 0.05, **p* < 0.1.

Figure 2 illustrates the periods *T*_int_ and *T*_*act*_ in relation to our survey period. In Figure A1 we add an arbitrary cutoff for our ‘close to’ group at *T* ^*D*^. In the first instance where the respondents last sex act was closer to Tabaski [*Date*_1_], $T_{1}^{D}$ is included in the ‘close to’ group. Where the last sex act falls after our cutoff [*Date*_2_], $T_{2}^{D}$ falls in our control group of sex acts.

**TABLE A8:** Effect of Last Sex Act being ‘close to’ Tabaski differentiated by ‘Those still to purchase an animal’ on Condom Use.

Variables	(1)	(2)	(3)	(4)	(5)	(6)	(7)	(8)
	*T^D^*	*T* ^*D*^	*T* ^*D*^	*T* ^*D*^	*T* ^*D*^	*T* ^*D*^	*T* ^*D*^	*T* ^*D*^
	< = 4 days	< = 5 days	< = 6 days	< = 7 days	< = 8 days	< = 9 days	< = 10 days	< = 11 days
Unbought animal * close to Tabaski * list	0.033	−0.699	−0.539	−0.461	−0.427	−0.316	−0.151	−0.165
	(1.021)	(0.493)	(0.355)	(0.338)	(0.290)	(0.284)	(0.251)	(0.241)
Close to Tabaski * list	−0.333	−0.076	−0.219	−0.259	−0.157	−0.171	−0.172	−0.047
	(0.311)	(0.244)	(0.193)	(0.189)	(0.165)	(0.153)	(0.150)	(0.155)
Unbought animal * list	−0.080	−0.048	−0.044	−0.047	−0.044	−0.058	−0.065	−0.058
	(0.090)	(0.090)	(0.091)	(0.092)	(0.094)	(0.095)	(0.096)	(0.097)
Not yet bought an animal	0.161**	0.140**	0.150**	0.148**	0.158**	0.162**	0.157**	0.156**
	(0.063)	(0.064)	(0.065)	(0.066)	(0.066)	(0.066)	(0.067)	(0.067)
Sensitive list	0.395*	0.397*	0.392*	0.400*	0.411*	0.406*	0.425*	0.410*
	(0.230)	(0.231)	(0.229)	(0.228)	(0.231)	(0.230)	(0.229)	(0.229)
Non‐sensitive list A	−0.349***	−0.347***	−0.342***	−0.340***	−0.341***	−0.342***	−0.342***	−0.343***
	(0.044)	(0.044)	(0.043)	(0.044)	(0.043)	(0.043)	(0.044)	(0.044)
New * list	−0.118	−0.075	−0.039	−0.032	−0.039	−0.044	−0.059	−0.093
	(0.106)	(0.107)	(0.109)	(0.109)	(0.111)	(0.113)	(0.113)	(0.115)
Delayed * list	0.057	0.057	0.078	0.076	0.071	0.071	0.061	0.064
	(0.163)	(0.163)	(0.160)	(0.160)	(0.160)	(0.161)	(0.162)	(0.163)
FSW age * list	0.009	0.008	0.008	0.008	0.008	0.008	0.008	0.008
	(0.005)	(0.005)	(0.005)	(0.005)	(0.005)	(0.005)	(0.005)	(0.005)
Risk aversion * list	−0.034	−0.045	−0.028	−0.027	−0.034	−0.034	−0.035	−0.037
	(0.060)	(0.060)	(0.059)	(0.059)	(0.060)	(0.059)	(0.060)	(0.060)
Constant	2.051***	2.057***	2.041***	2.040***	2.023***	2.032***	2.027***	2.040***
	(0.152)	(0.153)	(0.152)	(0.152)	(0.151)	(0.151)	(0.150)	(0.150)
Observations	810	810	810	810	810	810	810	810
*R* ^2^	0.240	0.241	0.243	0.244	0.242	0.241	0.238	0.237
Effect of Tabaski on celebrators								
Linear combination	−0.299	−0.775*	−0.758**	−0.72**	−0.584**	−0.488*	−0.323	−0.212
*p*‐value	0.76	0.075	0.014	0.013	0.021	0.056	0.136	0.292
Double list experiment	Yes	Yes	Yes	Yes	Yes	Yes	Yes	Yes
Key controls	Yes	Yes	Yes	Yes	Yes	Yes	Yes	Yes
*T* ^*act*^ < 90 only	Yes	Yes	Yes	Yes	Yes	Yes	Yes	Yes
Number of FSWs	405	405	405	405	405	405	405	405
FSWs ’yet to purchase’ and in the ’close to’ group	2	8	12	13	16	17	23	26

*Note*: Robust standard errors in parentheses. Specification 1 with sub‐groups (Equation (7)) with the last sex act within *T* ^*D*^ days of Tabaski defining ‘close to’ Tabaski interacted with the sub‐group of those ‘who intend to but have not yet bought an animal’ equaling 1 and those who have already bought an animal, those with no intention of and those not celebrating Tabaski equaling 0. The top row is the parameter of interest, *β*_7_. Linear combination is the effect of being ‘close to’ Tabaski for celebrators. Columns from left to right are separate regressions. Data of double list experiment with FSW level clustered standard errors. The sample is limited to those who have sex acts within the last 90 days and regressions include the key controls of FSW age, new FSW to the survey, delayed interview and risk aversion. Covariates without list treatment are included but not reported for brevity. There are no sex acts within 3 days for both sub‐groups. Beyond *T* ^*D*^ < = 13 + *days* the key parameter estimates remain similar and statistically non‐significantly different from zero.

****p* < 0.01, ***p* < 0.05, **p* < 0.1.

**TABLE A9:** Effect of Tabaski on log Price of Last Sex Acts.

	(1)	(2)
Variables		Log price
*T^D^*	0.001	0.000
	(0.004)	(0.003)
Sex act number		0.119***
		(0.036)
FSW age	0.004	0.000
	(0.004)	(0.004)
New FSW to the survey	0.121	0.002
	(0.097)	(0.085)
Interview was delayed	0.290**	0.236*
	(0.146)	(0.136)
Risk aversion	−0.164***	−0.133***
	(0.052)	(0.045)
Constant	9.010***	8.961***
	(0.231)	(0.206)
Observations	345	661
*R* ^2^	0.044	0.041
Key controls	Yes	Yes
Sex‐act FE	No	Yes
Number of FSWs	345	363

*Note*: Standard errors in parentheses. Regression specification 4 (Equation (10)) with logged price as dependent variable and the number days between sex act and Tabaski as the continuous shock variable. A continuous type of *T* ^*D*^. The top row contains the parameter of interest *β*_3_. Model 1 is cross sectional model of last sex acts only. Model 2, a pooled OLS including both last and penultimate sex acts. Controls used were FSW age, being new to the survey, if the interview was delayed, risk aversion and a measure for intensity ‐ the typical number of clients seen in 7 days. The sample was limited to having both sex acts within 28 days and the top and bottom 2.5% were dropped. Model 2 errors clustered at the FSW level.

****p* < 0.01, ***p* < 0.05, **p* < 0.1.

**TABLE A10:** Effect of Tabaski on the last client's characteristics.

Variables	(1)	(2)	(3)	(4)
	Risk of HIV	As clean as typical	As rich as typical	Client age
*T^D^* Continuous variable	0.015	0.006**	0.005*	−0.018
	(0.014)	(0.003)	(0.003)	(0.037)
FSW age	−0.005	−0.005	−0.004	0.648***
	(0.016)	(0.003)	(0.003)	(0.042)
Typical number of clients in 7 days	0.052**	0.011**	0.001	−0.000
	(0.024)	(0.004)	(0.005)	(0.064)
New FSW to the survey	−0.418	−0.007	0.043	−0.571
	(0.353)	(0.066)	(0.069)	(0.949)
Interview was delayed	−0.406	0.038	0.133	0.459
	(0.537)	(0.101)	(0.106)	(1.458)
Risk aversion	−0.215	−0.069*	−0.003	0.020
	(0.188)	(0.036)	(0.037)	(0.506)
Last client was a regular	0.444	0.003	−0.147**	0.886
	(0.363)	(0.070)	(0.073)	(0.980)
Constant	1.154	0.490***	0.556***	18.071***
	(0.869)	(0.167)	(0.175)	(2.337)
Observations	361	349	335	358
*R* ^2^	0.034	0.058	0.028	0.448
Key controls	Yes	Yes	Yes	Yes

*Note*: Standard errors in parentheses. Regression specification 4 (Equation (10)) column title as the dependent variable and the number days between sex act and Tabaski *T* ^*D*^ as the continuous shock variable. Risk of HIV is a rating out of 10 that the FSW believes the client has HIV. As clean and as rich is an indicator that the client is different from that FSWs typical client. Client age is in years. The top row contains the parameter of interest *β*_3_. Controls used were FSW age, being new to the survey, if the interview was delayed, risk aversion and a measure for intensity ‐ the typical number of clients seen in 7 days. The sample was limited to having both sex acts within 28 days.

****p* < 0.01, ***p* < 0.05, **p* < 0.1.

## APPENDIX B List Experiment Validation

We test the assumptions of the list experiment as per (Lépine et al., 2020; Treibich & Lépine, 2019) and Blair and Imai (2012).

### A Randomization

The randomization for the lists was done during the 2017 wave of the survey and the same lists and randomization was maintained in the 2020 wave. For new entrants to the survey we allocated alternately for each new FSW interviewed by each enumerator. Table B1 shows randomization was successful.

### B Design Effects

Rows 5 and six of Table B2 shows there is no design effects in list A, but there is some evidence of a design effect in Row six of List B because no‐one agreed with 0 statements in the control group. The treatment group has only 2 agree with 0 statements and for all other number of statements there is no sign of a design effect. An alternative method for testing the design effects detailed in Chuang et al. (2021) finds there is no significant difference between estimates from the A and B lists. Additionally my own test of design effect by including an interaction term of the list treatment variable and list assignment (list A or list B) in the double list regression which will capture the differential effect of answering the sensitive statement depending on which control list was received. This also gives a non‐significant difference suggesting no overall concerns for design effects.

### C Floor and Ceiling Effects

Table B2 summarizes the floor and ceiling effects and the design effects assumptions. Rows 1 to 4 show there is no floor or ceiling effect for List A. The control group have a low number of responses (<10%) for 0 and 3. List B on the other hand, Row 3 of List B shows there is some sign of a ceiling effect (23% for agreement with 3 statements) and so privacy could be compromised for some respondents in this list treatment group. When examining the control statements for list B, we note that COVID could have increased the likelihood of all 3 being agreed with. For the first ”*The majority of my clients are Senegalese*.”, designed for most to agree with, but with reduced international travel due to COVID, the chance of local clients increases. For the second and third ”I usually spend the whole night with my client.” & ”I usually solicit clients by phone.”, COVID has closed bars and clubs throughout the data collection period meaning a common place for finding clients and performing sex acts with clients is closed off potentially pushing solicitation to phones and encourages sex acts to take place at home increasing the chance of overnight stays. However, social desirability bias in our case acts to over‐report condom use so a ceiling effect will not influence the FSWs propensity to be dishonest and should not affect our results.

**TABLE B1:** Test of randomization with descriptive statistics.

Stat.	Means			Diff. A versus *B*
Variable	All	List A	List B	*p‐value*
Beauty (mean/10)	5.8	5.8	5.8	0.0
Age (years)	39.0	38.4	39.6	1.2
Risk preference (0‐2)	0.8	0.8	0.8	−0.0
Number of children	3.1	3.1	3.0	−0.1
Dependency ratio	1.4	1.5	1.3	−0.2
New FSW to survey (%)	35.8	35.6	36.0	0.4
Marital status: Never married (%)	21.0	20.9	21.1	0.1
Marital status: Married (%)	0.8	0.4	1.1	0.8
Marital status: Divorced or separated (%)	70.4	70.8	70.1	−0.6
Marital status: Widowed (%)	7.8	7.9	7.7	−0.2
Education: No education (%)	51.4	49.4	53.3	3.8
Education: Koranic only (%)	0.8	1.2	0.4	−0.8
Education: Elementary (%)	26.1	26.5	25.7	−0.8
Education: Middle? (%)	13.0	15.0	11.1	−3.9
Education: Secondary (%)	8.6	7.5	9.6	2.1
Education: University (%)	0.2	0.4	0.0	−0.4
Registered (%)	46.9	44.7	49.0	4.4
Has a second job (%)	37.2	34.4	39.8	5.5
Logged typical earnings (all, CFAF)	11.7	11.7	11.7	0.1
Wealth: Poorest (%)	32.5	32.8	32.2	−0.6
Wealth: Poor (%)	10.5	11.9	9.2	−2.7
Wealth: Neither (%)	16.9	16.2	17.6	1.4
Wealth: Rich (%)	21.0	18.2	23.8	5.6
Wealth: Richest (%)	19.1	20.9	17.2	−3.7
Both parents are alive (%)	22.4	21.3	23.4	2.0
Both parents are dead (%)	32.1	29.2	34.9	5.6
Has some available savings (%)	23.5	23.7	23.4	−0.3
Age of last client (years)	43.7	43.0	44.4	1.4*
Last client was a regular client (%)	80.7	84.6	77.0	−7.6**
Likely last client had HIV (%)	4.1	4.7	3.4	−1.3
Client consumed alcohol at last sex (%)	10.4	11.6	9.2	−2.3
Price was negotiated(%)	46.1	45.8	46.4	0.5
Last sex took place outside (%)	7.6	8.3	6.9	−1.4
Duration of last sex (mins)	12.7	13.1	12.4	−0.7
Last sex included fellatio (%)	16.2	15.5	16.9	1.4
Last sex included anal sex (%)	0.6	0.4	0.8	0.4
Stayed the night at last sex‐act (%)	7.6	7.6	7.7	0.1
Last client was rich (%)	5.4	4.0	6.9	2.9
Observations	514	253	261	514

**TABLE B2:** Test of design, floor and ceiling effects.

Estimated	Source	Number of reported items (*y*)						
Proportions		N	0	1	2	3	4	Sum
Senegal								
List A								
Row 1	Treatment list	261	0.004	0.034	0.544	0.391	0.027	1
Row 2	Pr(*Y*_*i*_ ≤ *y*|*T*_*i*_ = 1)		0.004	0.038	0.582	0.973	1	
Row 3	Control list	253	0.0079	0.344	0.565	0.083		
Row 4	Pr(*Y*_*i*_ ≤ *y*|*T*_*i*_ = 1)		0.008	0.352	0.917	1.000	1	
Row 5	Row 4 ‐ Row 2 (<0)		0.004	0.313	0.335	0.027		0.679
Row 6	Row 2 ‐ Row 4 (*y* − 1) (<0)		‐	0.030	0.231	0.056	0.000	
List B								
Row 1	Treatment list	253	0.008	0.036	0.289	0.577	0.091	1
Row 2	Pr(*Y*_*i*_ ≤ *y*|*T*_*i*_ = 1)		0.008	0.043	0.332	0.909	1	
Row 3	Control list	261	0.000	0.149	0.617	0.234		1
Row 4	Pr(*Y*_*i*_ ≤ *y*|*T*_*i*_ = 1)		0.000	0.149	0.766	1.000	1	
Row 5	Row 4 ‐ Row 2 (<0)		−0.008	0.106	0.434	0.091		0.623
Row 6	Row 2 ‐ Row 4 (*y* − 1) (<0)		‐	0.043	0.183	0.143	0.000	
