# Supplementary material for: Trading HIV for sheep: Risky sexual behavior and the response of female sex workers to Tabaski in Senegal
Source: Health Econ. 2023 Nov 2;33(1):153–93. doi: 10.1002/hec.4756 (PMC10952657; doi:10.1002/hec.4756)
Supplement: Supplementary file 1 — Supporting Information S1 [file HEC-33-153-s001.pdf]

Supplementary Materials

Figure 1: Single-sided List Experiment Effect of Tabaski on Condomless Sex

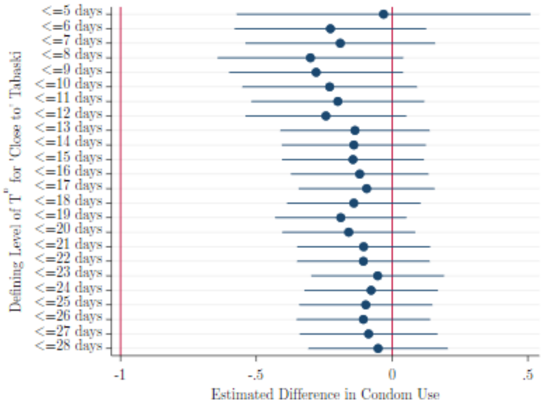

(a) List A without controls

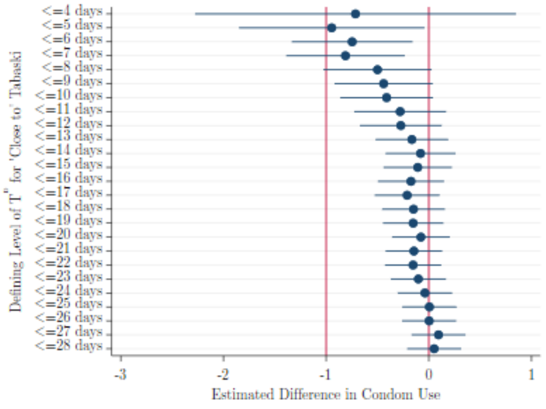

(b) List B without controls

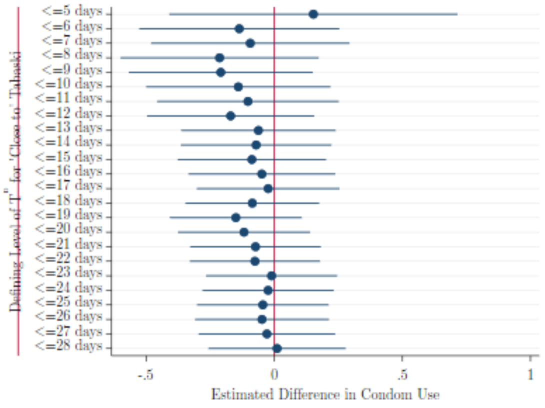

(c) List A with controls

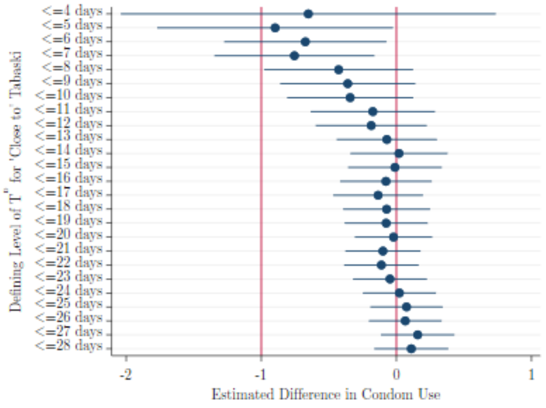

(d) List B with controls

Supplementary Figure 1 estimates the primary analysis using the single-sided version of the list experiment. When comparing to the results in Figure 3 of the main paper, it is clear the coefficients of both single-single list experiments lie within the ranges of each other, and importantly within the range of double list-experiment presented in the main paper. Results tables for these figures are available on request.

Table 1: Relationship between FSW household dependency ratio and days between last sex and Tabaski

VARIABLES	(1) Dependency Ratio	(2) Dependency Ratio
Days between last sex act and Tabaski	0.009*	0.008
	(0.095)	(0.147)
Registered		0.065
		(0.761)
FSW Age		-0.019
		(0.155)
Gneezy-Potter risk preference /2		0.133
		(0.301)
Time preference		-0.027
		(0.911)
Number of children		0.355***
		(0.000)
Intensity - typical number of clients in 7 days		0.015
		(0.414)
Marital Status: Married		0.298
		(0.830)
Marital Status: Divorced or separated		0.436*
		(0.097)
Marital Status: Widowed		0.588
		(0.200)
New FSW to survey		-0.190
		(0.437)
Logged typical earnings (all sources)		-0.018
		(0.908)
Both parents are alive		0.285
		(0.240)
Both parents are dead		0.301
		(0.233)
Constant	1.098***	0.481
	(0.000)	(0.801)
Observations	411	408
R^2	0.007	0.309
FSW covariates	No	Yes
Wealth covariates	No	Yes
Client/sex-act covariates	No	No
$T^{act} < 90$ only	Yes	Yes
Number of FSWs	411	408

pval in parentheses

*** p<0.01, ** p<0.05, * p<0.1

Model 1 regresses a binary variable equal 1 if individual celebrates Tabaski, model 2 and 3 if an individual intends to buy an animal for the celebration. All unreported variables are not statistically significant at 1% or 5% levels. Unreported marital status reference category - never married. Unreported education categories, reference category - no education. Unreported wealth categories, reference category - middle 20%. The sample is limited to those who have sex acts within the last 90 days. **Gneezy-Potter Risk preference** is an investment game to determine the risk aversion of individuals with values of 0 to 2. **FSW household dependency ratio** is the ratio of children and under 65's to adults in the FSWs household. **Time preference** is a percentage of those who prefer money today instead of twice as much in one weeks time.

Table 2: Robustness - Effect of Last Sex Act being 'close to' Tabaski on Condom Use Prevalence excluding FSWs with delayed interviews

VARIABLES	(1) T^D <= 4 days	(2) T^D <= 5 days	(3) T^D <= 6 days	(4) T^D <= 7 days	(5) T^D <= 8 days	(6) T^D <= 9 days	(7) T^D <= 10 days	(8) T^D <= 11 days
Close to Tabaski * List	-0.325	-0.416	-0.375**	-0.395**	-0.274*	-0.268*	-0.230*	-0.128
	(0.508)	(0.259)	(0.177)	(0.170)	(0.147)	(0.139)	(0.131)	(0.131)
Close to Tabaski	-0.338	0.080	0.145	0.103	0.130	0.097	0.110	0.042
	(0.256)	(0.172)	(0.122)	(0.120)	(0.115)	(0.106)	(0.102)	(0.100)
Sensitive list	0.328	0.363	0.339	0.343	0.361	0.347	0.358	0.345
	(0.230)	(0.229)	(0.227)	(0.227)	(0.228)	(0.227)	(0.227)	(0.228)
Non-sensitive List A	-0.346***	-0.346***	-0.340***	-0.340***	-0.338***	-0.339***	-0.338***	-0.339***
	(0.045)	(0.045)	(0.045)	(0.045)	(0.045)	(0.045)	(0.045)	(0.045)
New * List	-0.095	-0.060	-0.027	-0.020	-0.033	-0.027	-0.035	-0.064
	(0.107)	(0.108)	(0.109)	(0.110)	(0.111)	(0.113)	(0.113)	(0.115)
FSW age * List	0.009*	0.009	0.009*	0.009*	0.008	0.009*	0.009	0.009*
	(0.005)	(0.005)	(0.005)	(0.005)	(0.005)	(0.005)	(0.005)	(0.005)
Risk aversion * List	-0.039	-0.049	-0.029	-0.027	-0.030	-0.032	-0.037	-0.038
	(0.060)	(0.060)	(0.060)	(0.060)	(0.060)	(0.060)	(0.060)	(0.060)
Constant	2.211***	2.199***	2.197***	2.198***	2.185***	2.194***	2.186***	2.195***
	(0.154)	(0.154)	(0.154)	(0.155)	(0.154)	(0.154)	(0.153)	(0.154)
Observations	752	752	752	752	752	752	752	752
R^2	0.227	0.226	0.227	0.228	0.225	0.225	0.225	0.223
Double list experiment	Yes	Yes	Yes	Yes	Yes	Yes	Yes	Yes
Key controls	Yes	Yes	Yes	Yes	Yes	Yes	Yes	Yes
T^{act} < 90 only	Yes	Yes	Yes	Yes	Yes	Yes	Yes	Yes
Number of FSWs	412	412	412	412	412	412	412	412
FSWs in the 'close to' group	4	17	34	36	47	53	60	65

Robust standard errors in parentheses
*** p<0.01, ** p<0.05, * p<0.1

Specification 1 with the last sex act within T^D days of Tabaski defining 'close to Tabaski'. The top row is the parameter of interest, β_3. Each column is a separate regression. Data of double list experiment with FSW level clustered standard errors. The sample is limited to those who have sex acts within the last 90 days and **excludes those with delayed interviews**. Regressions include the key controls of FSW age, new FSW to the survey and risk aversion. Covariates without list treatment are included but not reported for brevity. There are no sex acts within 3 days of $T^D <= 11 + days$ the key parameter estimates remain similar and statistically non-significantly different from zero.

Table 3: Robustness - Effect of Last Sex Act being 'close to' Tabaski on Condom Use Prevalence excluding FSWs with delayed interviews

	(1)	(2)	(3)	(4)	(5)	(6)	(7)	(8)
	T^D	T^D	T^D	T^D	T^D	T^D	T^D	T^D
VARIABLES	<= 4 days	<= 5 days	<= 6 days	<= 7 days	<= 8 days	<= 9 days	<= 10 days	<= 11 days
Close to Tabaski * List	-0.450	-0.514**	-0.453***	-0.468***	-0.363***	-0.342***	-0.308**	-0.222*
	(0.565)	(0.259)	(0.168)	(0.160)	(0.135)	(0.127)	(0.120)	(0.119)
Close to Tabaski	-0.275	0.155	0.184	0.148	0.169	0.139	0.150	0.094
	(0.254)	(0.167)	(0.113)	(0.110)	(0.103)	(0.095)	(0.092)	(0.090)
Sensitive list	0.613***	0.631***	0.649***	0.653***	0.653***	0.656***	0.657***	0.646***
	(0.046)	(0.046)	(0.047)	(0.047)	(0.048)	(0.049)	(0.050)	(0.050)
Non-sensitive List A	-0.337***	-0.337***	-0.331***	-0.331***	-0.328***	-0.329***	-0.328***	-0.326***
	(0.046)	(0.045)	(0.045)	(0.045)	(0.045)	(0.045)	(0.045)	(0.046)
Constant	2.112***	2.102***	2.089***	2.092***	2.084***	2.085***	2.081***	2.088***
	(0.040)	(0.040)	(0.041)	(0.041)	(0.041)	(0.041)	(0.042)	(0.042)
Observations	752	752	752	752	752	752	752	752
R^2	0.217	0.217	0.219	0.221	0.218	0.218	0.217	0.215
Double list experiment	Yes	Yes	Yes	Yes	Yes	Yes	Yes	Yes
Key controls	No	No	No	No	No	No	No	No
$T^{act} < 90$ only	Yes	Yes	Yes	Yes	Yes	Yes	Yes	Yes
Number of FSWs	412	412	412	412	412	412	412	412
FSWs in the 'close to' group	4	17	34	36	47	53	60	65

Robust standard errors in parentheses
*** p<0.01, ** p<0.05, * p<0.1

Specification 1 with the last sex act within T^D days of Tabaski defining 'close to Tabaski'. The top row is the parameter of interest, β_3. Each column is a separate regression. Data of double list experiment with FSW level clustered standard errors. The sample is limited to those who have sex acts within the last 90 days and **excludes those with delayed interviews**. Covariates without list treatment are included but not reported for brevity. There are no sex acts within 3 days of $T^D <= 11 + days$ the key parameter estimates remain similar and statistically non-significantly different from zero.

Table 4: Determinants of Tabaski celebrators

VARIABLES	(1) Tabaski Celebrators	(2) Animal Purchases	(3) Animal Purchases
Registered	0.027	0.084	0.079
	(0.469)	(0.161)	(0.141)
FSW Age	0.000	0.003	0.002
	(0.987)	(0.349)	(0.483)
Gneezy-Potter risk preference /2	-0.055**	-0.098***	-0.108***
	(0.016)	(0.007)	(0.001)
Time preference	-0.008	0.100	0.088
	(0.851)	(0.131)	(0.147)
Number of children	0.000	0.000	-0.001
	(0.973)	(0.986)	(0.891)
Dependency ratio	-0.002	0.012	0.014
	(0.832)	(0.357)	(0.263)
New FSW to survey	-0.075**	-0.072	-0.097*
	(0.045)	(0.228)	(0.070)
Intensity - typical number of clients in 7 days	-0.004	-0.002	-0.002
	(0.259)	(0.771)	(0.706)
Marital Status: Married	0.131	-0.273	-0.153
	(0.591)	(0.440)	(0.659)
Marital Status: Divorced or separated	0.067	-0.075	-0.022
	(0.152)	(0.321)	(0.733)
Marital Status: Widowed	0.091	-0.126	-0.074
	(0.257)	(0.328)	(0.521)
Logged typical earnings (all sources)	0.051*	0.117***	0.122***
	(0.055)	(0.006)	(0.002)
Both parents are alive	-0.062	-0.024	-0.057
	(0.143)	(0.720)	(0.345)
Both parents are dead	-0.040	0.033	0.008
	(0.362)	(0.634)	(0.900)
Constant	0.151	-0.831	-0.873*
	(0.655)	(0.107)	(0.063)
Observations	409	341	402
R^2	0.182	0.133	0.163
FSW covariates	Yes	Yes	Yes
Wealth covariates	Yes	Yes	Yes
Includes non-tabaski celebrators	Yes	No	Yes
$T^{act} < 90$ only	Yes	Yes	Yes
Number of FSWs	409	312	402

pval in parentheses

*** p<0.01, ** p<0.05, * p<0.1

Model 1 regresses a binary variable equal 1 if individual celebrates Tabaski, model 2 and 3 if an individual intends to buy an animal for the celebration. All unreported variables are not statistically significant at 1% or 5% levels. Unreported marital status reference category - never married. Unreported education categories, reference category - no education. Unreported wealth categories, reference category - middle 20%. The sample is limited to those who have sex acts within the last 90 days. **Gneezy-Potter Risk preference** is an investment game to determine the risk aversion of individuals with values of 0 to 2. **FSW household dependency ratio** is the ratio of children and under 65's to adults in the FSWs household. **Time preference** is a percentage of those who prefer money today instead of twice as much in one weeks time.

Figure 2: Coefficient Graph of Parameter [β_3] of Models Estimated with dependency ratio as a key control

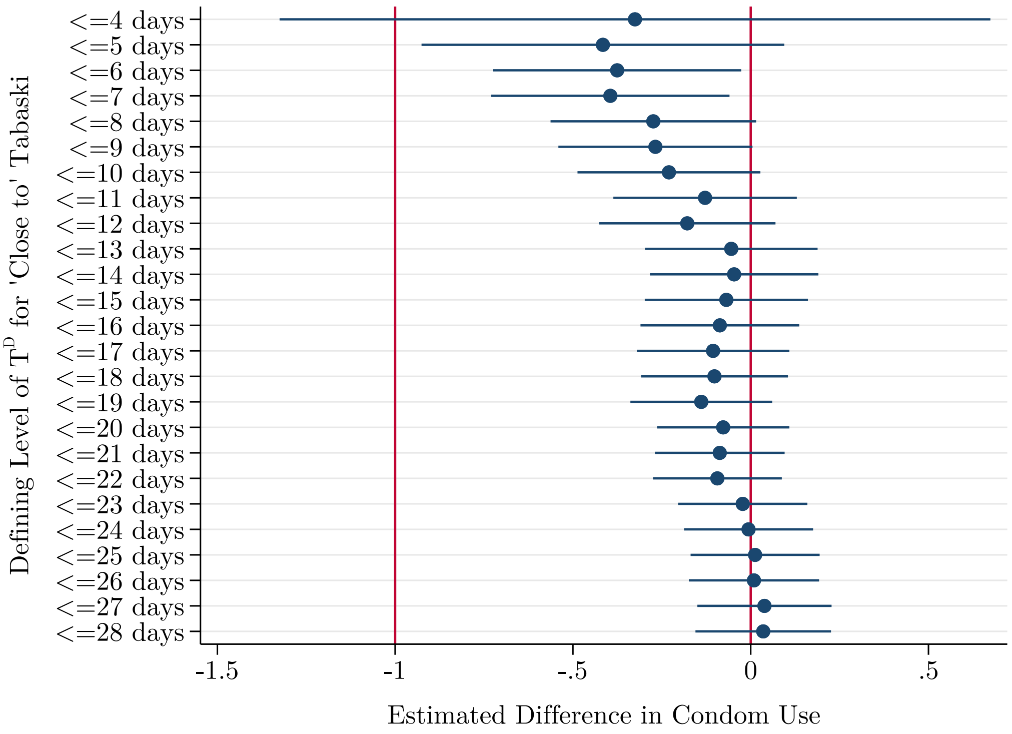

The typical control variable indicating those with delayed interviews has been replaced by the FSWs household dependency ratio. All other key controls are included, namely, FSW age, new FSW to the survey and risk aversion. Only those with sex acts with 90 days of Tabaski are included. Results table for this figure is available on request.

Figure 3: Coefficient Graph of Parameter [β_3] of Models Estimated with days since last sex as a key control

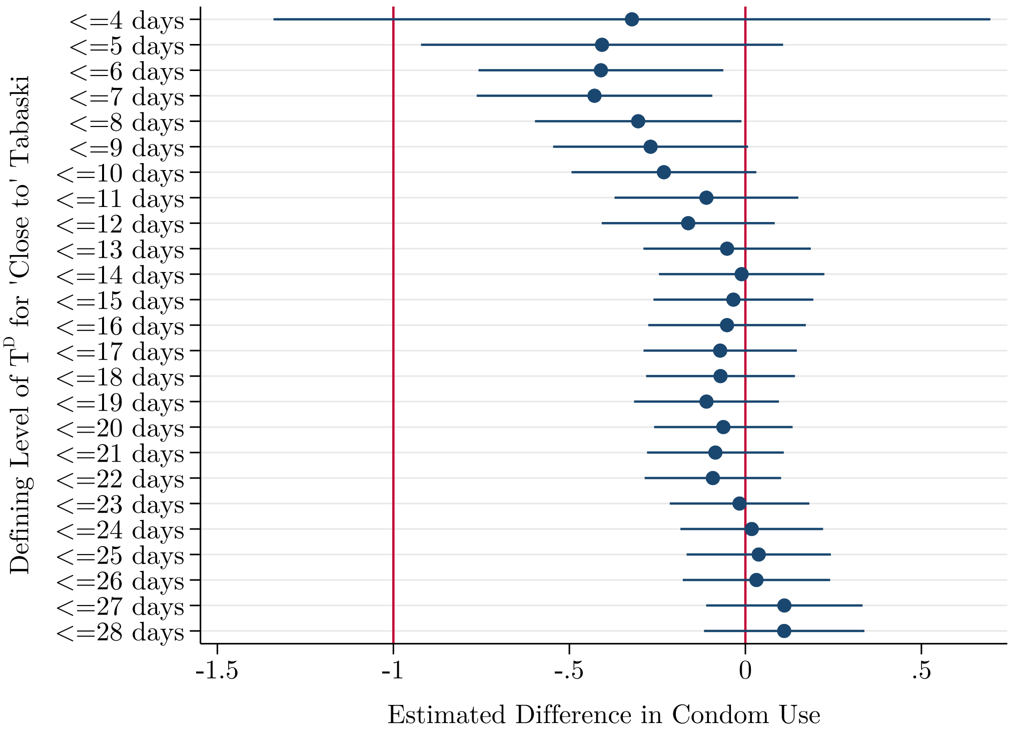

Key controls of FSW age, new FSW to the survey and risk-aversion are included alongside the continuous variable representing the number of days between the last sex act and Tabaski to a maximum of 90. Results table for this figure is available on request.

Figure 4: Coefficient Graph of Parameter [β_3] of Models Estimated using the sub-sample of Tabaski celebrators only

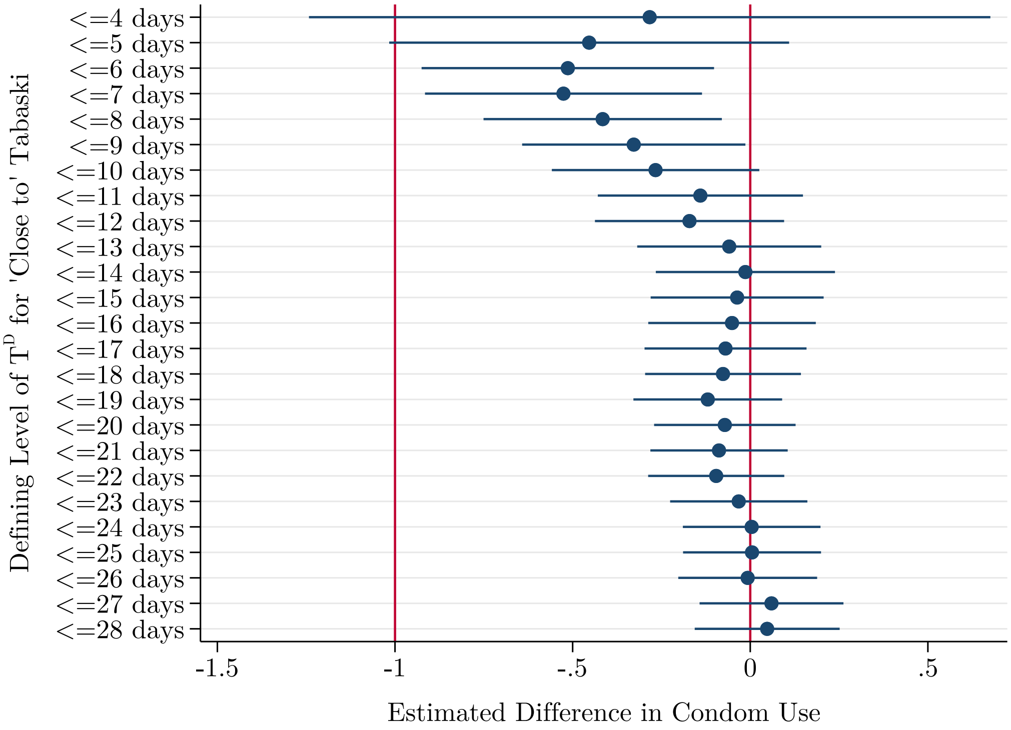

Key controls of FSW age, new FSW to the survey and risk-aversion are included alongside the continuous variable representing the number of days between the last sex act and Tabaski to a maximum of 90. Results table for this figure is available on request.

Figure 5: Coefficient Graph of Parameter [β_3] of Models Estimated using only FSWs with sex acts within 7 days of the interview

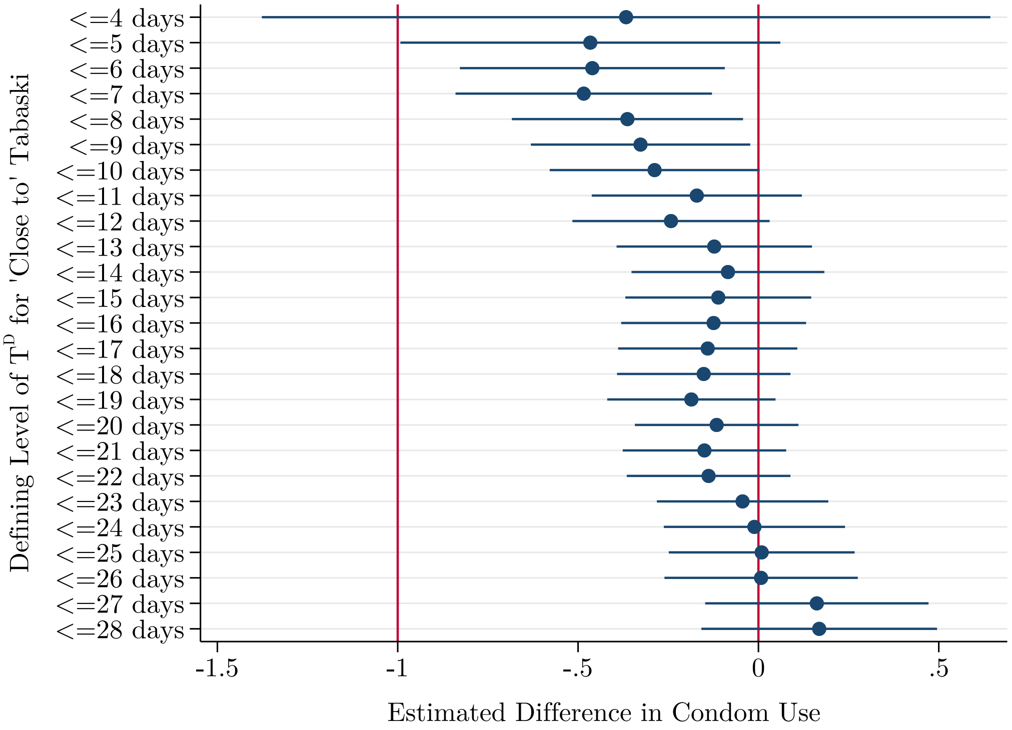

Key controls of FSW age, new FSW to the survey and risk-aversion are included alongside the continuous variable representing the number of days between the last sex act and Tabaski to a maximum of 7. Results table for this figure is available on request.

Table 5: Weekend effect on condom use

VARIABLES	(1)<br>Sex at the Weekend	(2)<br>Sex at the Weekend	(3)<br>Sex at the Weekend
Weekend * List	-0.099	-0.010	0.153
	(0.288)	(0.913)	(0.228)
Sensitive list	0.660***	0.663***	0.602***
	(0.000)	(0.000)	(0.000)
Indicator for sex at the weekend	0.163**	0.125*	-0.030
	(0.011)	(0.062)	(0.730)
Constant	1.856***	1.863***	1.951***
	(0.000)	(0.000)	(0.000)
Observations	824	750	474
R^2	0.176	0.202	0.199
$T^{act} < 90$	Yes	Yes	Yes
$T^{act} < 7$	No	No	Yes
$^{D} < 7$	Yes	No	No
Number of FSWs	412	375	237

Robust pval in parentheses

*** p<0.01, ** p<0.05, * p<0.1

Table 6: Difference in condom use between occasional and regulars

VARIABLES	(1)<br>Condom use	(2)<br>Condom use
Occasional client * List	-0.031	-0.004
	(0.757)	(0.966)
Occasional client	-0.012	-0.033
	(0.858)	(0.628)
Sensitive list	0.657***	0.339*
	(0.000)	(0.091)
lista	-0.332***	-0.338***
	(0.000)	(0.000)
Constant	2.072***	2.152***
	(0.000)	(0.000)
Observations	1,028	1,028
R^2	0.243	0.259
Key controls	No	Yes
$T^{act} < 90$	Yes	Yes
Number of FSWs	514	514

Robust pval in parentheses
*** p<0.01, ** p<0.05, * p<0.1
